# Looking for Typical Traits in Monovarietal VOOs According to Their Phenolic Composition

**DOI:** 10.3390/foods13213425

**Published:** 2024-10-27

**Authors:** Maria Giovanna Molinu, Pierfrancesco Deiana, Sandro Dettori, Luca Mercenaro, Giovanni Nieddu, Antonio Dore, Nicola Culeddu, Mario Santona

**Affiliations:** 1 Istituto di Scienze delle Produzioni Alimentari (ISPA), CNR, Traversa La Crucca 3, Loc. Baldinca, Li Punti, 07040 Sassari, Italy; mariagiovanna.molinu@cnr.it (M.G.M.); antonio.dore@cnr.it (A.D.); 2 Dipartimento di Agraria, Università degli Studi di Sassari, Viale Italia 39, 07100 Sassari, Italy; sdettori@uniss.it (S.D.); mercenaro@uniss.it (L.M.); gnieddu@uniss.it (G.N.); msantona@uniss.it (M.S.); 3 Istituto di Chimica Biomolecolare (ICB), CNR, Traversa La Crucca 3, Loc. Baldinca, Li Punti, 07040 Sassari, Italy; nicola.culeddu@icb.cnr.it

**Keywords:** Coratina, Frantoio, *Olea europaea* L., oleacein, oleocanthal, Pearson correlation, varietal characterization

## Abstract

Due to its high sensitivity to numerous variability sources, it is hard to define the typicity of a monovarietal virgin olive oil (VOO) according to its phenolic profile. In this study, we aimed to identify the features of phenolic composition that are persistent and minimally affected by variability sources, making them potential varietal markers. We separately analyzed three databases of monovarietal VOO phenolic compositions, determined by liquid chromatography, from three different cultivars. The first database was produced from the original data of the Bosana cultivar. The other two were obtained through a systematic analysis of scientific literature on Coratina and Frantoio cultivars. Several statistical tools, including coefficient of variability, correlations, and linear regression models, were used to find recurring proportions or ratios unaffected by variability sources suitable to define typical varietal traits. Some proportions between molecules, mostly within the same phenolic class, remain constant. Strong correlations between (i) flavonoids were observed in Bosana and Frantoio VOOs (R^2^ = 0.87 and 0.77, respectively), (ii) oleacein-oleocanthal (Bosana, R^2^ = 0.81) (iii) oleuropein aglycon-ligstroside aglycon (Frantoio, R^2^ = 0.88), and (iv) lignans (Coratina, R^2^ = 0.84). These traits could be useful tools for defining the typicity of monovarietal VOOs.

## 1. Introduction

Virgin olive oil (VOO) is the main lipid source of the Mediterranean diet. It is widely known for important benefits to human health, which are related to the high ratio of monounsaturated fatty acids and the presence of minor compounds [1]. Among them, preventing free radical damage and oxidative stress, polar phenols have been indicated as the principal responsible of VOO health activity [2]. Over the last years, the knowledge of its health and organoleptic properties favored the increase in VOO demand. This conscious consumption of high quality and healthy olive oils goes accompanied by the growing interest in territory of origin and related tradition, as well as on specific monovarietal VOOs, which are characterized by unique chemical and sensory profiles [3,4]. In this framework, the seeking of what makes typical a monovarietal VOO, its distinctive features in terms of sensory profile and chemical characteristics, is an actual concern for all the stakeholders of the olive oil sector. The concept of typicity, usually in accordance with that of terroir, is related to those attributes, both in terms of presence and/or abundance, which are common within most of the samples of a same variety [5,6]. As well as being observed in the vinery sector, sensory attributes are the principal character apt to define the typical VOO varietal traits [6,7,8]. Additionally, fatty acids, sterols, and terpenes have been demonstrated to be very useful to discriminate VOOs according to cultivars [8,9,10]. Indeed, for instance, the genetic factor is predominant in determining fatty acids and sterol profile, whilst the influence of external factors such as oil extraction technologies or ageing is marginal [1].

On the other hand, phenolic compounds, although contributing to sensory attributes such as bitterness and pungency [11], else being equal the variety, are affected by numerous factors, which can be defined as sources of variability, that make it hard to define a typical phenolic profile or concentration for a specific variety [1,12]. For instance, classifying cultivars as “poor” or “rich” sources of phenols might be misleading: from the same cultivar, we can produce VOO with a total phenolic amount lower than 100 mg kg^−1^ but also higher than 700 mg kg^−1^ [1].

Since phenolic compounds are strictly related to the plant’s natural defense mechanisms protecting tissues from oxidation processes, abiotic (drought, salinity, or extreme temperatures) and biotic stressors (such as insects, fungal or bacterial diseases) may induce both phenolic synthesis and phenolic depletion [13,14]. Consequently, the agronomical practices related to these stress factors (e.g., irrigation, fertilization, soil management, and pest control) can modify phenolic composition and amount. Water availability during fruit growth is inversely related with the buildup of phenolic compounds in olive fruits [15]. This is clear when water deficit occurs before pit hardening [15]. Indeed, according to Alagna et al. [16], the expression of the genes putatively related to phenolic biosynthesis was activated at the earliest stages of maturation. However, during oil synthesis, the relationship between abiotic stress (drought or thermal) and phenolic composition are quite controversial and variable according to the variety, planting system, and tree age [13,15,17]. In the context of a deficit irrigation trial performed on a young super-high-density orchard (cv. Arbequina), Garcia et al. [13] observed that severe water stress conditions during later phases of fruit development caused lower phenolic concentrations. Authors hypothesized a strong role of the oxidative enzymatic activity of the stressed fruits in promoting phenolic degradation that in turn protected olive tissues from oxidative stress.

The sensitivity of the individual molecules to agronomical treatments or environmental stresses is often different. For instance, vanillin, phenolic acids, and lignans were found to have low sensitivity to different irrigation treatments [13]. Angilè et al. [18] highlighted that the phenolic compounds of VOO varied according to the type of water resource used for irrigation. According to the authors, the use of reclaimed water, characterized by higher salinity, induced a secoiridoids accumulation as a stress response to high salt levels. In addition, some authors observed that olives from canopy areas with increased sun exposure reported higher phenolic amounts [19]. On the other hand, it is well known that increased fruit irradiation speeds up the maturation process [20], which, in turn, is responsible for the progressive polyphenolic reduction [16,21]. Among the individual phenolic compounds, the secoiridoids are those that show the greatest depletion due to the enzymatic activity of β-glucosidase and polyphenoloxidase. Otherwise, flavonoids, lignans, and phenolic alcohols may increase or decrease during fruit ripening, but to a lesser extent compared to the secoiridoids [16].

Further agronomical practices such as foliar applications with kaolin and salicylic acid during the summer season, aimed at reducing heat stress and preventing olive fly attacks, contributed to an increase in VOO phenolic concentrations [22]. Nitrogen fertilization, at an optimal range level of 50–100 mg/kg of nitrogen available in soil, may contribute to improving the phenol amount; conversely, higher levels might induce opposite effects [23]. According to the same authors, only oleocanthal seems to be unconnected with N availability.

In addition to the environmental and agronomical factors, the variables related to the extraction technologies, such as time, oxygen levels, and temperatures during malaxation, and type of decanter and crusher, strongly affect the VOO phenolic profile [24,25]. For this reason, extraction parameters are calibrated according to the variety and fruit characteristics. For instance, moisture and fatty acid composition of fruits may affect the activity of endogenous enzymes, such as methylesterase and β-glucosidase, which are mainly responsible for the levels oleuropein and ligstroside derivatives in VOO [26,27]. Moreover, the phenolic molecules evolve differently during storage, both according to their initial concentration and to the variety [28,29].

In addition, the great number of analytical techniques and methods proposed for identification and quantification of phenolic compounds makes it hard a direct data comparison and causes further uncertainty [12].

In the field of varietal and geographical origin characterization based on phenolic composition, numerous chemometric techniques both supervised (discriminant analyses) or unsupervised (cluster analyses) are largely used, reporting high accuracy and efficiency [4,12,30]. These multivariate approaches were able to identify those molecules which variability most contributed to discriminate cultivars between each other. However, those findings are still partial, since they remain true only within the varieties considered and to the samples studied per each cultivar, which usually resulted in a relatively inadequate number, not representative enough for a given variety.

It seems that the identification of typical/characteristic VOO according to the phenolic profile varietal features is still in progress and probably needs novel approaches. Thus, throughout the present study, we aimed to identify those features of phenolic composition which are persistent and minimally affected by variability sources, thus potentially able to be defined as typical of a variety. To this purpose, three databases of monovarietal VOO’s phenolic composition, determined by liquid chromatography, coming from three different cultivars, were analyzed separately.

The first database was produced by original data belonging to the cultivar Bosana. The VOO samples were collected from the typical growing areas of Sardinia (Italy), accounting for different variability factors: soil and climate conditions related to different growing areas and seasons, extraction technology, and storage period. The role of the three factors on a phenolic profile of Bosana VOOs was investigated through univariate and multivariate statistics. The two further databases were produced through a systematic analysis of available scientific literature on two of the most widespread Italian varieties: Coratina and Frantoio. These latter datasets, besides accounting environmental and technological variability, accounted also the variability related to the different analytical methods adopted by the different authors to identify and quantify phenolic compounds. Using the statistical approaches detailed below (see Section 2), we aimed to identify aspects of the phenolic composition that remain unaffected by the high variability of data included in the databases.

## 2. Materials and Methods

### 2.1. Bosana Database

The choice of the Bosana as the variety object of the original database of the present study was motivated by its relevance at the Italian and international level [1,25]. It is the most widespread Sardinian autochthonous olive cultivar [1,31]. Its principal attitude is oil production, the typical sensory attributes of which are grass, artichoke thistle, and fresh almond, together with medium-higher bitter and spicy sensations [31,32]. According to the Italian monovarietal database [33], the sensory profile of Bosana VOOs belongs to the same typology of other relevant Italian varieties such as Carolea, Peranzana, Maurino, Nocellara Messinese, Biancolilla, and Ottobratica.

Sixty-three VOO samples from the cultivar Bosana were analyzed. Oils were kindly supplied by local farmers during the 2015, 2016, 2018, and 2020 (26, 21, 6, and 10 samples, respectively) harvest seasons. Oils were produced during the months of November and December. VOOs came from the following municipalities of North and West Sardinia: Alghero (*n* = 9), Sassari—Sorso—Sennori (*n* = 20), Ittiri (*n* = 13), and Oristano (*n* = 21). These areas cover a wide variability of soils (e.g., alluvial and calcareous soils, trachytes, lithosols, and terre rosse) and different bioclimatic conditions, from the hot and arid coasts (Alghero and Oristano) to the subhumid hilly inland areas (Ittiri) [34,35]. The dataset included VOO produced by local industrial mills, which differ for several technological aspects such as three-phase and two-phase decanters, and presence or absence of the centrifugal separator. Samples were provided by producers at different storage periods; consequently, we classified VOOs as fresh (30 samples between 0 and 3 months after production) and stored (33 samples between 4 and 7 months after production). Samples were processed for phenolic extraction soon after delivery.

### 2.2. Analysis of Phenolic Compounds

#### 2.2.1. Chemicals and Reagents

For the quantification of phenolic compounds, the following analytical standards, purchased from Sigma-Aldrich (Milano, Italy and St. Louis, MO, USA), were used: hydroxytyrosol (≥98%), tyrosol (≥98%), oleacein (≥98%), oleocanthal (≥98%), vanillin (≥99%), vanillic acid (≥97%), *p*-coumaric acid (≥98%), pinoresinol (≥95%), luteolin (≥98%), and apigenin (≥95%). Methanol for HPLC (≥99.9%, gradient grade, suitable for HPLC) and trifluoroacetic acid, suitable for HPLC, ≥99%, were provided by Sigma Co (St. Louis, MO, USA). Acetonitrile (≥99.9%, gradient grade, suitable for HPLC) was purchased by ChemLab (Zedelgen, Belgium). A Milli-Q system (Millipore Corporation, Billerica, MA, USA) was used to prepare ultrapure water (H_2_O).

#### 2.2.2. Sample Preparation

The phenolic compounds were extracted according to the method described by the International Olive Council [36], modified as described by Deiana et al. [37]. Briefly, 4 g of oil sample were dissolved in 5 mL of methanol/water (80:20, *v*/*v*). The mixture was shaken for 30 min and then centrifuged for 5 min at 3360 g. The polar supernatant was separated. The extraction process was performed twice, and the extracted polar fraction was filtered through 0.45 μm PVDV filters. Samples were stored at −80 °C until analysis.

#### 2.2.3. RP-HPLC-DAD Analysis

Analysis of phenolic compounds was performed on an Agilent 1100 LC System (Agilent Technologies, Palo Alto, CA, USA) equipped with a quaternary pump (G1311A), degasser, column thermostat, auto-sampler (G1313A), diode array detector (G1315 B, DAD), and a Luna C18 column (250 × 4.6 mm, 5 µm) from Phenomenex (Torrance, CA, USA) with a security guard cartridge (4 × 2 mm). The flow rate was set at 1 mL/min, column temperature 30 °C, and injection volume was 20 μL. Chromatographic separation was carried out as reported by Deiana et al. [37]. Phenolic compounds were detected at 280 and 320 nm, identified according to retention time of a mixture of standards and quantified using calibration curves with five points of dilution specific to the respective standards (mg L^−^^1^). The phenolic compounds, secoiridoids (oleacein, oleocanthal, oleuropein, and ligstroside aglycon) and acetoxypinoresinol, were identified by liquid chromatography–mass spectrometry (LC-MS) analysis as described in our previous work [37]. Quantification of secoiridoids and 1-acetoxypinoresinol was performed using the calibration curves of oleuropein and pinoresinol. Results are expressed as mg of phenolic compounds per kg of oil.

### 2.3. Systematic Review on Frantoio and Coratina Cultivars VOO Phenolic Compounds

The following codes were run on Scopus and Google Scholar research engines, looking on title, abstract, keywords, and main text:

- Coratina AND “olive oil” AND phenol AND HPLC
- Frantoio AND “olive oil” AND phenol AND HPLC

Only original research papers that determined phenolic compounds through liquid chromatography were included. The values, expressed as mg kg^−^^1^, of hydroxytyrosol, tyrosol, oleacein, oleocanthal, oleuropein aglycon, ligstroside aglycon, 1-acetoxypinoresinol, pinoresinol, luteolin, and apigenin were included in the dataset. These ten phenolic molecules were selected since they are the most representative in VOO, as well as those mostly reported in literature. The variability factors (for instance extraction methods, area of origin, growing year, and agronomical practices) considered in each study were reported in specific columns (see Supplementary Material, Tables S3 and S4).

### 2.4. Statistical Analysis

Data were expressed as absolute values (concentration, mg kg^−^^1^) and relative values (percentage of the total sum of phenolic compounds analyzed by RP-HPLC-DAD), and analyzed by some basic statistics (median, standard deviation, coefficient of variability, correlations, and linear regression models) aimed at finding those constant, recurring proportions or ratios that could be considered typical for Bosana VOO. Similarly, the datasets obtained from the literature review for Coratina and Frantoio monovarietal VOOs were analyzed through descriptive statistics (e.g., normal distribution and data distribution with regression line and confidence intervals), Pearson correlation, and linear regression model analysis between the phenolic molecules.

Moreover, using the Bosana dataset, the sample’s information about growing area, year, and storage period, their influence on the phenolic profile related ratios, was evaluated. The percentage of variance related to the three factor was measured. Each of them was settled as random factors of linear mixed effect models (lme4 package of R-Studio version 4.1.3) [38]. This statistical approach was adopted due to the unbalanced nature of the experimental plan. Therefore, the difference between means of categories among each factor (typical of analysis of variance, ANOVA) could not be estimated consistently; on the other hand, considering factors as random, throughout mixed effect models, the role of each factor was estimated in terms of related variance. In other words, we only assess how much phenolic molecules vary according to the considered factors, not how much a phenolic molecule differs according to a specific factor. Moreover, in order to identify the phenolic individual molecules, groups of them, or associated ratios, less related to the three factors, and thus more suitable as indicators of varietal typicity, orthogonal projection to latent structures discriminant analysis (OPLS-DA) models were performed with the use of SIMCA-P software version 13.0 (Umetrics AB, Umea, Sweden). This multivariate was selected since it provides a simple and clear interpretation of variable importance throughout the variable importance on projection (VIP) parameter: the higher is the value, the higher is the role of the variable in discriminating among the model classes (in this case, represented by the levels of the factors year, area, and storage period) [28,29]. Models were validated according to the permutations test (significance level was set at α < 0.05) [31].

## 3. Results

### 3.1. Bosana VOO Phenolic Composition

Twelve individual phenolic compounds were identified and quantified, as shown in Table 1, where the median content of each phenolic compound, expressed as absolute (mg kg^−^^1^) and relative (%) values, minimum and maximum values, standard deviation, and coefficient of variation (CV), were reported. A representative RP-HPLC-DAD chromatogram is available in Supplementary Material (Figure S1).

As expected, secoiridoids were the most represented phenolic class (91.8%). If compared to the other classes, the secoiridoids’ relative amount, despite the wide overall variability, showed a very low coefficient of variation (CV = 3.2). This suggests a strong link between secoiridoid fraction and the genetic factor. Similarly, also the relative fraction of the health claim compounds (HC), which includes both secoiridoids and phenolic alcohols, was consistent (94.9%, CV = 1.9). In this study, 41 of the 63 Bosana samples (around 65%) achieved the limit required by the EU regulation [39], which is 250 mg kg^−^^1^.

Following this order, oleacein, oleocanthal, oleuropein aglycon, and ligstroside aglycon were the phenolic molecules with the highest average concentration. About 70% of oleacein values were within the range of 30–130 mg kg^−^^1^, whereas the same percentage of oleocanthal values ranged between 18 and 95 mg kg^−^^1^. More than 80% of the samples reported higher levels of oleacein with respect to oleocanthal, and the ratio between the two molecules was, on average, 1.4 (±0.4).

Phenolic alcohols represented the second class of molecules in Bosana VOO, on average of 3.3%. Among them, a predominance of tyrosol was observed and the concentration ranged between 2.8 and 29.0 mg kg^−^^1^. On the other hand, in 70% of samples, hydroxytyrosol concentration clustered between 2 and 4 mg kg^−^^1^. The only lignan found was 1-acetoxypinoresinol (3.8–32.7 mg kg^−^^1^).

The flavonoids luteolin and apigenin reported relatively low variability and a normal-like distribution, as well as vanillin. These three compounds were those with the lowest CV levels (41.0%, 41.4%, and 33.9%, respectively). The luteolin levels were always higher than apigenin ones, and the ratio between them was 1.6 (±0.3) on average (Table S2), and about the 70% of the oil samples showed values within the range of 1.3–1.8. The *p*-coumaric and vanillic acids were present at lower concentrations and not always quantifiable in Bosana VOOs.

**Figure 1:**
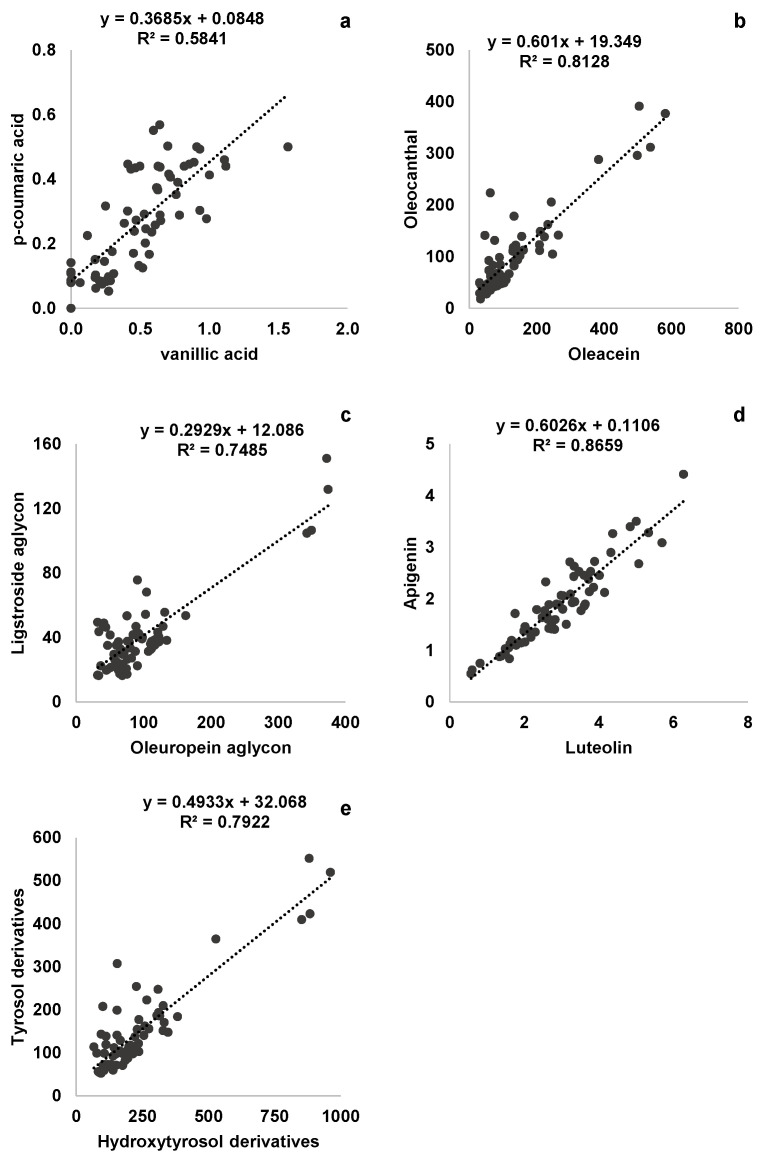
Main correlations and regression equations identified within the phenolic profile (mg kg^−^^1^) of Bosana VOOs: *p*-coumaric acid–vanillic acid (**a**), oleocanthal–oleacein (**b**), ligastroside aglycon–oleuropein aglycon (**c**), apigenin–luteolin (**d**), tyrosol derivatives (sum of tyrosol, oleocanthal, and ligstroside aglycon)–hydroxytyrosol derivatives (hydroxytyrosol, oleacein, and oleuropein aglycon) (**e**).

Among the 1035 Pearson correlations performed, the molecules belonging to the same phenolic class reported the strongest relationships. According to the R value obtained and to the physiological and analytical implication, five correlations were selected to perform the respective linear regression models (Figure 1, for equations details see Table S2). High and positive linear correlation were obtained between oleacein and oleocanthal (R^2^ = 0.81). Other strong correlations were obtained between the two flavonoids, luteolin and apigenin (R^2^ = 0.87), the two aglycon forms of oleuropein and ligstroside (R^2^ = 0.75), and between the phenolic acids *p*-coumaric and vanillic (R^2^  =  0.58). Finally, the relationship between tyrosol derivatives and hydroxytyrosol derivatives (respectively: the sum of tyrosol, oleocanthal, and ligstroside aglycon, and the sum of hydroxytyrosol, oleacein, and oleuropein aglycon) was strong (Figure 1e).

### 3.2. Frantoio and Coratina VOO Phenolic Composition

The Coratina VOOs were largely studied during the last 25 years; indeed, the phenolic profile was described by 49 research papers available in the Scopus and Google Scholar archives (the complete raw dataset is reported in Table S3), from which 179 observations were extrapolated. The phenolic alcohols and secoiridoids were the molecules most frequently reported (from 110 observations of ligstroside aglycon to 171 observations reporting tyrosol amounts). On the other hand, less information was available for flavonoids (only 60 observations) and lignans (76 and 68 observations, respectively, for 1-acetoxypinoresinol and pinoresinol). The latter phenolic group was those with the relatively lower variability (60% and 46%, respectively). Oil extraction techniques and innovative technologies were the variability factors principally investigated when involving Coratina variety.

**Figure 2:**
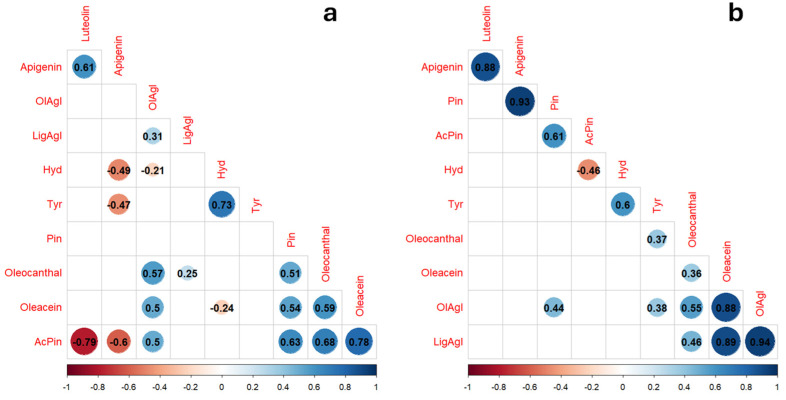
Correlation matrix between the data acquired for 10 phenolic molecules, respectively, regarding Coratina VOOs (**a**) and Frantoio VOOs (**b**). Only the R values of significative Pearson correlations (*p*-value < 0.01) are reported in the figures.

**Figure 3:**
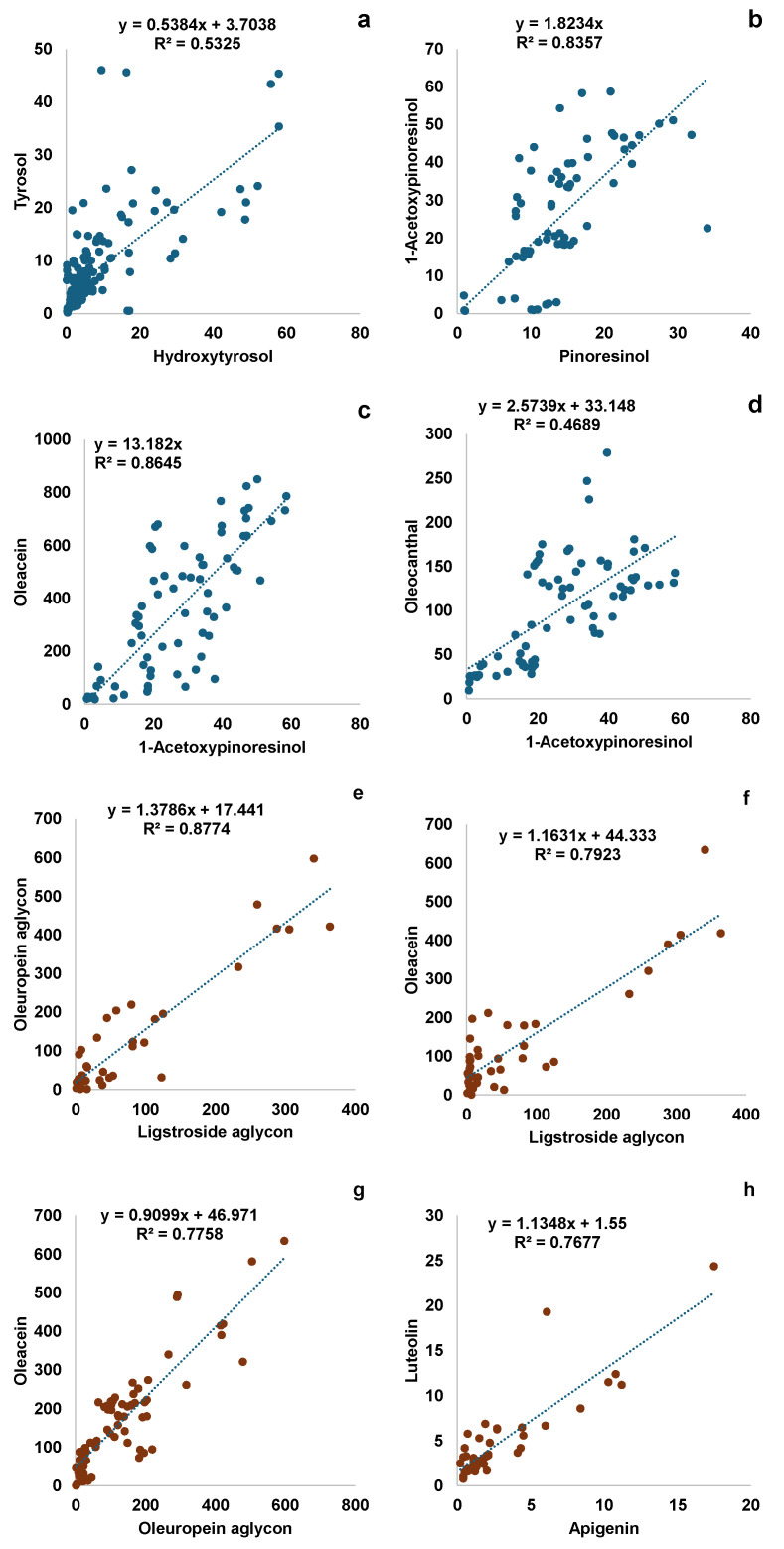
Scatter plot reporting the linear regression model equations between individual phenolic molecules, potentially useful as varietal markers, for Coratina (**a**–**d**) and Frantoio (**e**–**h**) VOOs.

In order to identify stable proportion between the 10 phenolic molecules, the dataset was analyzed, producing a Pearson’s correlation matrix (Figure S2). As reported in Figure 2a, among the 45 possible pairwise correlations, 19 of them obtained a significance level (*p*-value < 0.01). According to the R value and to the number of observations available, four couples of phenols were selected as potentially suitable for indicators of typicality in Coratina VOOs, and a linear regression model was performed for each of them. Figure 3a–d reports the equations for the four Coratina regression models. The regression models between the two lignans (1-Acetoxypinoresinol vs. Pinoresinol), and Oleacein vs. 1-Acetoxypinoresinol, provided equations with intercept set to 0 and achieved the highest R^2^ values (0.84 and 0.87, respectively).

The dataset about the Frantoio VOO phenolic composition was obtained from 27 research papers available in the Scopus and Google Scholar archives, which provided 74 observations, together with 9 observations provided by the author’s archive (Table S4 and Figure S3). Similarly to Coratina, the phenolic alcohols and secoiridoids were the phenolic molecules mostly investigated, but lignans were those that reported the lowest levels of variability (CV = 48.9% and 68.8%, respectively, for 1-acetoxypinoresinol and pinoresinol).

The correlation analysis of the Frantoio dataset highlighted the strongest relationship between the molecules of the same phenolic families (Figure 2b). It is worth noting the high correlation between the two aglycons (ligstroside and oleuropein) and the two flavonoids (Figure 3e,h). Indeed, the corresponding regression models reported high R^2^ values (0.88 and 0.77, respectively). In addition, the regression models between oleacein and both oleuropein aglycon and ligstroside aglycon were significant, reporting high R^2^ values.

### 3.3. The Role of Variability Factors on Phenolic Composition

The influence of the factors (i) year, (ii) growing area, and (iii) storage, on the single phenolic molecules, group of them, and the identified stable ratios was measured in terms of variance through linear mixed effect models analysis using the Bosana database. The results (Table S5) reveal that, among the factors considered, the growing year was the one which showed the highest variability related to the phenolic composition, particularly to phenolic acids and phenolic alcohols.

On the other hand, storage period was principally related to the flavonoids, acetoxypinoresinol, and oleacein variability. Another relevant piece of information provided by the mixed model analysis is that the variability observed in the proposed ratios (specifically: flavonoids, aglycons, and phenolic acids) was almost equally distributed by the three factors. It is worth noting that most of the restricted maximum likelihood (REML) models performed showed high error values; this could be partially related to other factors that were not possible to separate. Indeed, due to its unbalanced nature, through the present case of study, it was not possible to separate agronomical and technological factors from the growing area.

**Figure 4:**
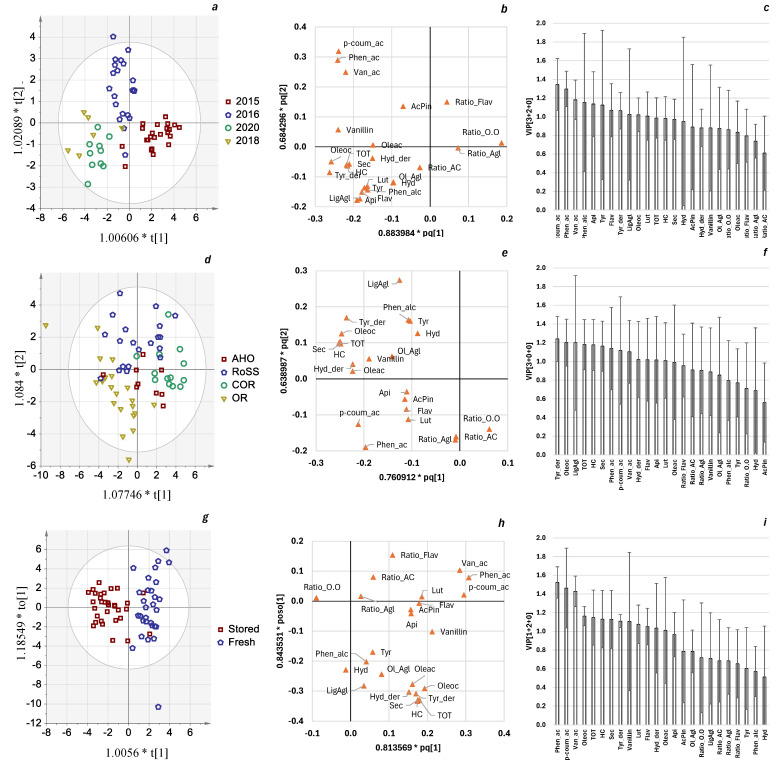
Summary plots of OPLS-DA models aimed at discriminating the Bosana VOOs according to different sources of variability: growing year (**a**–**c**), area of origin (**d**–**f**), and storage time (**g**–**i**). Figures (**a**,**d**,**g**) represent the observation scores colored by respective classes. Figures (**b**,**e**,**h**) represent the model loadings, showing the relationships among the X variables of the model. Figures (**c**,**f**,**i**) represent the variable importance on projections (VIPs) of the model, describing the importance of the variable to explain X and correlate with Y. Variable’s abbreviations: AcPin = 1-acetoxypinoresinol; Api = apigenin; Flav = flavonoids; HC = health claim; Hyd = hydroxytyrosol; Hyd_der = sum of Hyd, Oleac, Ol_Agl; LigAgl = ligstroside aglycon; Lut = luteolin; Ol_Agl = oleuropein aglycon; Oleac = oleacein; Oleo = oleocanthal; p-coum_ac = *p*-coumaric acid; Phen_ac = phenolic alcohols; Phen_alc = phenolic alcohols; Ratio_AC = Van_ac/p-coum_ac; Ratio_Agl = Ol_Agl/LigAgl; Ratio_Flav = Lut/Api; Ratio_O.O = Oleac/Oleoc; Sec = secoiridoids; TOT = sum of phenols; Tyr = tyrosol; Tyr_der = sum of Tyr, Oleoc, Lig_agl.; and Van_ac = vanillic acid.

Since the analysis of variance is univariate, through this approach, it is not possible to achieve a wider view about the relationship between the variability factors and phenolic profile and to compare the weight of each variable (in this case phenols) in defining the factor classes. For this reason, a multivariate approach by OPLS-DA analysis was adopted. Through the same analysis, we tried to identify the phenolic features less related to the three factors, and thus more suitable as varietal indicators. OPLS-DA models results are summarized in Figure 4. In line with the REML model’s overall results, the year and storage OPS-DA models obtained better performances in terms of goodness of fit (R^2^X = 0.82 and R^2^X = 0.65, respectively, Table S6) and predictive ability (Q^2^ = 0.54 and Q^2^ = 0.69, respectively, Table S6). Looking at Figure 4a,b, we can see that the 2018 and 2020 seasons were charachterized by a higher phenolic concentration, specifically secoiridoids. Conversely, 2016 stood out for phenolic acids in higher amounts, whilst 2015 showed the lowest oleocanthal, oleacein, and vanillin concentrations. With regard to storage factor (Figure 4g–i), it is interesting to observe a uniform decreasing behavior of phenolic compounds from fresh to aged VOO samples; on the other hand, the four ratios proposed changed marginally between the two classes.

According to the loading plots and VIP values shown in Figure 4 (Figure 4b and Figure 4c, respectively), most of the phenolic classes contributed to the growing season variability, except for the lignans, represented in this study by 1-acetoxypinoresinol (Figure 4c). Low VIP values, below 1, of the four phenolic ratios were reported in the three OPLS-DA models (Figure 4c,f,i), indicating that those variables were not relevant for class discrimination.

## 4. Discussion

As expected, the results show a wide range of values, justifiable by the large number of sources of variability accounted in both original (Bosana) and literature (Coratina and Frantoio) datasets. In the case of a meta-analysis-like database, as for the Coratina and Frantoio cases, it is interesting to underline the presence of a further source of variability: the different analytical approaches adopted by each research group, which might contribute to increase the range of absolute values related to a specific phenolic molecule, even without precluding the identification of the typical varietal traits.

In terms of total phenolic content, our findings on Bosana VOOs (153.0–1510.8 mg kg^−1^) encompass the results reported by other authors about the same cultivar [25,37,40,41,42].

As is generally common for almost all VOOs, secoiridoids were the principal biophenolic molecules [1,25,43]. In the case of Bosana, the most representative was oleacein, followed by oleocanthal, and the aglycon forms of oleuropein and ligstroside. These findings are in line with previous literature that referred to this Sardinian cultivar [25,37,40,44], although in some cases, data were obtained through different methods of phenolic determination [25,44]. Oleacein was the principal phenolic molecule also in Frantoio and Coratina (Tables S3 and S4), followed by oleuropein aglycon and then oleocanthal.

The large range of values of tyrosol and hydroxytyrosol generally observed can be attributed principally to different degrees of ripeness, postharvest treatments, and storage conditions. The first factor may cause opposite effects depending on variety [24]. On the other hand, during storage, the concentration of phenolic alcohols increases because of the progressive hydrolysis of secoiridoids. For this reason, together with the presence of oxidized forms of secoiridoids, aged VOOs might be recognized by high values of phenolic alcohols [28,29].

Literature reports contrasting data about the lignans occurrence in Bosana VOOs. Some authors reported the presence of only 1-acetoxypinoresinol [37,44]. Oppositely, other authors reported the presence of only pinoresinol [42] or both lignans [40,41]. With respect to other national and international varieties, such as Semidana, Coratina, Frantoio, Piqual, and Arbequina, Bosana VOOs contain generally low levels of lignans [37,45,46]. Being that these compounds are relatively stable to the oil extraction process, López-Biedma et al. [46] proposed to use the levels of pinoresinol over 1-acetoxypinoresinol as varietal markers. Further explanation of lignans stability in VOOs might be due to their main location in olive stones and twigs [46], which have tissues that are less sensitive to environmental variability. This aspect was confirmed by the literature data collected on Coratina and Frantoio (Table S3 and Figure S2), which revealed a lower variability of lignans concentration and a normal-like distribution (Table S4 and Figure S3). In this regard, it is worth noting the stable proportion between the amounts of both lignans in Coratina VOOs, which could be considered a distinctive trait of this variety.

That of lignans observed in the Coratina phenolic profile is one of the numerous stable proportions within phenolic molecules we observed in all the three varieties studied. The close relationships observed within the same phenolic class are supported by a physiological point of view. Indeed, the individual molecules belonging to the same phenolic class, except for hydroxytyrosol and tyrosol, share the same biosynthetic pathway [16]. Phenolic acids, flavonoids, and lignans are the products of the phenylpropanoid pathway based on the activity of the enzyme L-phenylalanine ammonia-lyase (PAL). Oleuropein seems to be the result of two biosynthetic pathways: (1) the plastidial 2-C-methyl-d-erythritol 4-phosphate (MEP) pathway, which is responsible for the biosynthesis of secologanin and oleoside-11-methyl ester, precursors of oleacein and ligstroside; and (2) the dopamine pathway, responsible for the biosynthesis of tyrosol [16]. However, the biosynthetic steps involving oleuropein derivatives, ligstroside, oleacein, oleocanthal, and aglycon derivatives are still not completely clear. According to Obied et al. [47], during oil extraction, the activity of β-glucosidase leads to the formation of aglycon forms of ligstroside and oleuropein, whilst methylesterase activity is responsible for the appearance of the corresponding demethylated forms (oleacein and oleocanthal). These enzymatic processes were confirmed indirectly by Miho et al. [25]. The authors, analyzing VOO samples from different varieties, reported a general strong correlation between the aglycon forms of oleuropein and ligstroside and between oleacein and oleocanthal. The latter correlation was also observed by Karkoula et al. [48]. Despite the use of different analytical methods (1H-NMR) and a different variety studied (Koroneiki), the authors reported a fitting value of the regression model between oleacein and oleocanthal (R^2^ = 0.78), which is very similar to our findings for Bosana (Figure 1b). The authors observed also that the ratio between the two molecules is almost stable and independent of harvest time and oil extraction conditions. In Koroneiki VOOs the ratio was in favor of oleocanthal, whilst the opposite was so in Bosana. According to these findings, the relationship between the two molecules seems to be strongly related to the genetic factor, thus its stable proportion could be considered a typical trait of the Bosana and Koroneiki phenolic profile; however, the value of the proportion is varietal-specific. On the other hand, looking at the other varieties investigated, it is worth noting the strong relationship between oleuropein aglycon and ligstroside aglycon and between oleacein and oleuropein aglycon typical of Frantoio. This latter pairwise correlation seems to be in line with previous literature findings [25,27].

The high correlation between luteolin and apigenin observed in Bosana and Frantoio monovarietal VOOs is common also in other cultivars and the respective amount and mutual relationship seems to be a varietal-specific feature [49]. The content of flavonoids has been found to be able to discriminate among monovarietal VOOs [21]. Moreover, some authors reported a relative stability in the concentration of luteolin and apigenin among different environmental conditions and along fruit maturation [21,42,45]. The low influence of such sources of variability on flavonoid concentration is probably the reason for the relatively low variation in the ratio, which is the most stable observed in this study (Table S1). For all these aspects, the proportion between luteolin and apigenin should be considered a very interesting and reliable tool for varietal characterization. Moreover, if compared for instance to secoiridoid and lignan analysis, also considering availability and cost of respective standards, flavonoid detection and quantification by LC is relatively easier, unambiguous, and cheaper.

Finally, the role of growing year, area of production, and storage time in phenolic composition was assessed using the dataset of Bosana VOOs. The predominance of growing year in other variability factors, as for instance, geographical origin or agronomical management, was previously described in literature for further VOO quality aspects such as fatty acid and organoleptic profiles [15,31]. The effect of growing area on Bosana VOOs was also the object of a recent study [50]. The authors observed that only the fatty acid composition was significantly related to the geographical origin (i.e., North-West Sardinia), whilst the minor fraction (volatile compounds, tocopherols, and phenolic compounds) was not able to discriminate the production areas. Authors hypothesized that the interannual variability contributed to the masking the effect of the other factor. Additionally, other authors, referring to Mediterranean climates at a regional scale, reported that the environmental variability, in terms of meteoclimatic conditions, was at first related to the growing season rather than geographical origin [31,51]. The OPLS-DA analysis confirmed the high susceptibility of phenolic compounds to seasonal meteoclimatic conditions, primarily summer heat and drought stresses, and to storage time [29,52,53]. Lignan, represented in this study by 1-acetoxypinoresinol, was the only phenolic class that was not affected by seasonal variability. Thanks to its relative stability to seasonality and oil extraction conditions, López-Biedma et al. [46] considered the levels of lignans strictly related to the genetic factor and suitable as VOO varietal markers. Regarding the relationship between storage and phenolic compounds, our results agree with previous literature, which described a degradation of phenolics during time and the secoiridoids as the individual molecules most involved in the process [53,54].

Focusing the attention on the four phenolic ratios, the multivariate analysis confirmed their relative independence against the investigated factors. Indeed, in all the three models, the ratios played a marginal role. These findings highlight that the interactions between the phenolic molecules pertaining to the same class of phenols are suitable tools for improving varietal characterization.

## 5. Conclusions

Throughout a detailed study of the VOO phenolic profile dataset, both from original research and from literature data, the present study aims to highlight that it is possible to obtain significant and useful information for monovarietal VOOs characterization. As extensively discussed, phenolic concentration and profile are highly variable. Identifying elements unaffected by this variability is advantageous for: (i) varietal characterization and identification; (ii) varietal selection based on specific qualitative traits desired by producers or the market; and (iii) providing further indirect insights into the physiology and biosynthesis of phenolic compounds. These findings should be considered as further tools that, together with what is already known of other VOO components (i.e., fatty acids, sterols, and volatile compounds), can contribute to enhance varietal and VOO knowledge.

Notwithstanding the high variability that characterizes the VOO phenolic profile, some proportions between molecules, most of which belong to the same phenolic class, are constant. The rate of these proportions and the molecules involved are different according to cultivar. The strong correlations between the two flavonoids luteolin and apigenin and the secoiridoids, in both Bosana and Frantoio VOOs, as well as phenolic alcohols and lignans in Coratina VOOs, represent varietal-specific traits: useful tools, together with sensory and volatile profile characterization, for defining monovarietal VOO’s typicity.

Moreover, the effectiveness of a meta-analysis-like database for the research of typical varietal traits related to phenolic compounds could be a valid alternative solution to overcome the common difficulty to acquire an adequate and representative number of samples. It is important to underline that the effort to recover high numbers of samples with related information on production would allow us to investigate better the influence of each source of variability in the VOO phenolic profile. With this regard, our findings on the Bosana database highlight the predominant role of seasonality in most phenolic molecules.

Further in-depth studies are necessary to improve the knowledge on the varietal-specific relationships between variability factor and VOO composition.

## Figures and Tables

**Table 1:** Concentration (mg kg^−^^1^) and relative fraction (%) of polar phenolic molecules in Bosana VOOs.

	Concentration (mg kg^−1^)					Relative Fraction (%)				
Variable	Median	Min	Max	SD ^1^	CV (%)	Median	Min	Max	SD	CV (%)
Hydroxytyrosol	2.8	0.6	9.2	1.5	47.4	1.0	0.3	3.1	0.6	60.7
Tyrosol	5.6	2.8	29.0	4.7	65.7	1.8	0.5	7.8	1.5	67.8
*p*-coumaric acid	0.3	0.0	1.0	0.2	67.6	0.1	0.0	0.3	0.1	72.6
Vanillic acid	0.5	0.0	1.6	0.3	65.2	0.1	0.0	0.8	0.1	90.8
Vanillin	0.4	0.1	0.8	0.2	33.9	0.1	0.0	0.4	0.1	47.5
1-Acetoxypinoresinol	10.0	3.8	32.7	5.1	45.2	3.3	1.0	7.4	1.6	37.7
Oleacein	89.1	30.4	582.9	116.8	89.3	30.5	13.1	48.1	7.7	24.7
Oleocanthal	68.2	18.0	391.4	77.1	79.2	23.7	11.7	47.1	6.7	28.1
Oleuropein aglycon	77.5	31.9	374.1	72.8	75.2	23.9	11.0	38.9	7.1	28.2
Ligstroside aglycon	36.6	16.4	151.0	24.6	61.0	10.6	5.3	26.4	4.1	36.6
Luteolin	2.8	0.6	6.3	1.2	41.0	0.9	0.2	1.9	0.4	47.6
Apigenin	1.8	0.6	4.4	0.8	41.4	0.6	0.1	1.2	0.3	43.5
Phenolic alcohols	8.6	4.5	34.9	5.7	55.0	2.9	0.7	10.0	2.0	60.3
Phenolic acids	0.8	0.0	2.1	0.5	58.8	0.2	0.0	1.1	0.2	81.8
Secoiridoids	280.1	132.8	1466.1	277.6	76.0	91.8	84.9	97.0	2.9	3.2
Flavonoids	4.8	1.1	10.7	1.9	40.4	1.5	0.4	3.1	0.7	45.3
^2^ Health claim sum	295.7	139.8	1481.2	278.4	74.1	94.9	89.6	98.0	1.8	1.9
Total phenolic content	311.2	153.0	1510.8	283.0	72.0					

^1^ SD = standard deviation; CV = coefficient of variation. ^2^ Health claim sum according to EC Reg no 432/2012 = sum of phenolic alcohols and secoiridoids.

## Data Availability

The original contributions presented in the study are included in the article/Supplementary Materials, further inquiries can be directed to the corresponding author.

## Supplementary Materials

The following supporting information can be downloaded at: https://www.mdpi.com/article/10.3390/foods13213425/s1, Figure S1: RP-HPLC-DAD representative chromatogram, at 280 nm (blue line) and 320 nm (red line), of Bosana VOO; Figures S2 and S3. Correlation plot of Coratina and Frantoio (respectively) phenolic compounds including correlation R values and respective significance, histogram and normal distribution, and data distribution with regression line and confidence intervals; Table S1: Details about the most stable ratios between individual phenolic molecules quantified by HPLC-DAD;. Table S2. Details about the linear regression models, potentially useful as varietal indexes, between individual phenolic molecules and phenolic groups; Table S3. Complete database of Coratina VOOs phenolic compounds; Table S4. Complete database of Frantoio VOOs phenolic compounds; Table S5. Results of variance analysis (%) from REML models for three factors of variability (years, growing area, and storage time) affecting VOO phenolic composition; Table S6. Autofit results for the Orthogonal Projections to Latent Structures Discriminant Analysis (OPLS-DA) models.
