# Supplementary material for: Looking for Typical Traits in Monovarietal VOOs According to Their Phenolic Composition
Source: Foods. 2024 Oct 27;13(21):3425. doi: 10.3390/foods13213425 (PMC11545162; doi:10.3390/foods13213425)
Supplement: Supplementary file 1 [file foods-13-03425-s001.zip › foods-3244235-supplementary.pdf]

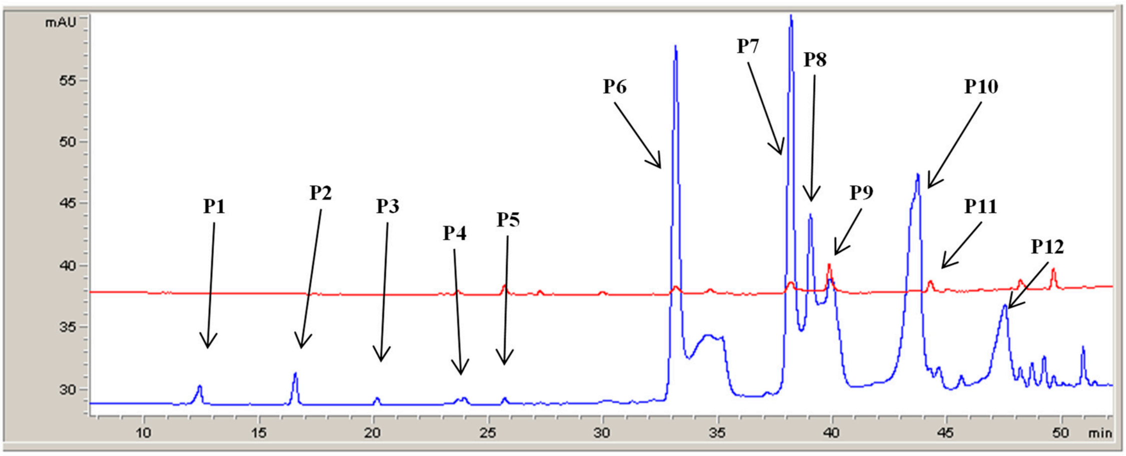

Figure S1. RP-HPLC-DAD representative chromatogram, at 280 nm (blue line) and 320 nm (red line), of Bosana VOO. P1 = hydroxytyrosol; P2 = tyrosol; P3 = vanillic acid; P4 = vanillin; P5 = p-coumaric acid; P6 = oleacein; P7 = oleocanthal; P8 = 1-acetoxypinoresinol; P9 = luteolin; P10 = oleuropein aglycon; P11 = apigenin; P12= ligstroside aglycon.

Table S1. Details about the most stable ratios between individual phenolic molecules quantified by HPLC-DAD.

Ratios	Average	SD	CV	Min	Max
p-coumaric acid/ Vanillic acid	0.57	0.29	51.13	0.19	1.89
Luteolin/ Apigenin	1.57	0.25	15.69	0.97	2.08
Oleacein/ Oleocanthal	1.38	0.43	31.35	0.28	2.35
Oleuropein Aglycon/ Ligstroside Aglycon	2.46	0.84	33.98	0.65	4.36

Table S2. Details about the linear regression models, potentially useful as varietal indexes, between individual phenolic molecules and phenolic groups.

Equation	R^2	Eq p-value	Constant p-value	X p-value
p-coumaric acid = 0.0848 + 0.3685 Vanillic acid	0.584	0.000	0.000	0.000
Oleocanthal = 19.349 + 0.601 Oleacein	0.813	0.000	0.004	0.000
Ligstroside aglycon = 12.086 + 0.2929 Oleuropein aglycon	0.749	0.000	0.000	0.000
Apigenin = 0.1106 + 0.6026 Luteolin	0.866	0.000	0.234	0.000
Tyrosol der = 32.068 + 0.4933 Hydroxytyrosol der	0.792	0.000	0.001	0.000

Table S3. Complete database of Coratina VOOs phenolic compounds.

Area	Year	Storage	Extraction	AgroManag	Ripening	Hyd	Tyr	Oleacein	Oleocanthal	Ol_Agl	LigAgl	AcPin	Pin	Luteolin	Apigenin	Reference
El Kef Tunisia	2014			irr1	1	2.10	6.66	144.83	106.65	128.81						Dabbou et al 2015 https://doi.org/10.1002/cbdv.201400142
El Kef Tunisia	2014			irr2	1	0.51	7.12	87.48	46.63	67.15						Dabbou et al 2015 https://doi.org/10.1002/cbdv.201400142
El Kef Tunisia	2014			irr3	1	0.13	6.30	60.53	36.70	54.43						Dabbou et al 2015 https://doi.org/10.1002/cbdv.201400142
El Kef Tunisia	2014			irr1	2	0.38	7.13	78.28	34.00	76.32						Dabbou et al 2015 https://doi.org/10.1002/cbdv.201400142
El Kef Tunisia	2014			irr2	2	0.16	9.10	70.61	27.74	61.50						Dabbou et al 2015 https://doi.org/10.1002/cbdv.201400142
El Kef Tunisia	2014			irr3	2	0.18	8.14	71.44	20.67	62.60						Dabbou et al 2015 https://doi.org/10.1002/cbdv.201400142
Apulia						3.80	7.60							0.90	0.90	Klikarova 2020 https://doi.org/10.1007/s00217-020-03484-1
Apulia						11.50	13.30							2.50	2.60	Klikarova 2020 https://doi.org/10.1007/s00217-020-03484-1
Apulia						9.00	11.70							1.50	1.40	Klikarova 2020 https://doi.org/10.1007/s00217-020-03484-1
Apulia						10.00	13.70							1.70	1.50	Klikarova 2020 https://doi.org/10.1007/s00217-020-03484-1
Apulia						3.10	4.80							3.70	3.20	Klikarova 2020 https://doi.org/10.1007/s00217-020-03484-1
Apulia						4.00	6.30							3.10	2.40	Klikarova 2020 https://doi.org/10.1007/s00217-020-03484-1
Apulia						8.40	14.10							3.00	2.80	Klikarova 2020 https://doi.org/10.1007/s00217-020-03484-1
Apulia						4.80	10.60							3.60	2.70	Klikarova 2020 https://doi.org/10.1007/s00217-020-03484-1
Apulia						2.70	6.60							2.90	3.00	Klikarova 2020 https://doi.org/10.1007/s00217-020-03484-1
Apulia						2.80	4.90							3.30	2.20	Klikarova 2020 https://doi.org/10.1007/s00217-020-03484-1
Apulia						4.30	4.50							2.10	1.30	Klikarova 2020 https://doi.org/10.1007/s00217-020-03484-1
Apulia						4.40	4.70							2.50	2.60	Klikarova 2020 https://doi.org/10.1007/s00217-020-03484-1
Brazil	2012					12.30	10.50	91.00	39.00	115.00	35.00	4.70	0.90	5.00	0.31	Ballus 2015 https://doi.org/10.1016/j.foodchem.2014.08.054
Apulia						9.90	4.40	485.00	128.00	112.60		23.20	17.70			Fabiani 2011 dx.doi.org/10.1021/jf201459u
Apulia	2005		traditional			3.10	14.90	365.20	92.80	101.20		41.10	8.40			Servili 2007 (10.1021/jf070600)
Apulia	2005		stoned			2.80	15.00	507.10	115.90	125.90		44.00	10.40			Servili 2007 (10.1021/jf070600)
Apulia	2013					4.30	2.63	18.67	24.64	5.60	2.52	2.93	13.52			Baiano 2016 https://doi.org/10.1007/s00217-016-2669-1
Apulia	2010		20°C O2=30kPa			2.20	3.70	106.00	37.90	79.20		19.10	14.50			Taticchi 2013 https://doi.org/10.1016/j.foodchem.2012.08.071
Apulia	2010		25°C O2=30kPa			0.90	1.70	128.00	44.30	106.10		19.20	15.90			Taticchi 2013 https://doi.org/10.1016/j.foodchem.2012.08.071
Apulia	2010		35°C O2=30kPa			2.20	2.20	176.70	83.60	135.70		18.20	15.40			Taticchi 2013 https://doi.org/10.1016/j.foodchem.2012.08.071
Apulia	2010		20°C O2=50kPa			2.00	2.60	47.10	28.00	44.80		18.20	14.60			Taticchi 2013 https://doi.org/10.1016/j.foodchem.2012.08.071
Apulia	2010		25°C O2=50kPa			1.00	1.60	55.40	34.70	50.00		18.40	13.70			Taticchi 2013 https://doi.org/10.1016/j.foodchem.2012.08.071
Apulia	2010		35°C O2=50kPa			1.00	2.50	68.30	41.80	64.60		18.40	14.30			Taticchi 2013 https://doi.org/10.1016/j.foodchem.2012.08.071
Apulia	2000		hammer			2.30	2.90	301.60	52.60	257.00						Servili 2002 https://doi.org/10.1002/1438-9312(200208)104:8<483::AID-EJLT483>3.0.CO;2-M
Apulia	2000		blade			1.10	3.80	366.60	67.00	269.40						Servili 2002 https://doi.org/10.1002/1438-9312(200208)104:8<483::AID-EJLT483>3.0.CO;2-M
Apulia	2014		25°C			5.10	10.00	505.80	123.70	235.30	22.70	44.50	23.80			Veneziani 2017 http://dx.doi.org/10.1016/j.foodchem.2016.10.067
Apulia	2014		25°C cooled			10.90	23.60	517.30	127.50	240.70	30.80	43.40	22.80			Veneziani 2017 http://dx.doi.org/10.1016/j.foodchem.2016.10.067
Apulia	2014		30°C			5.10	5.60	730.40	136.90	355.40	25.10	46.50	22.70			Veneziani 2017 http://dx.doi.org/10.1016/j.foodchem.2016.10.067
Apulia	2014		30°C cooled			7.30	7.80	741.40	138.00	368.10	27.40	47.70	21.10			Veneziani 2017 http://dx.doi.org/10.1016/j.foodchem.2016.10.067
			ctrl			1.51	1.39	253.00	36.05	112.35	29.65					Servili 2000 10.17660/ActaHortic.2002.586.116
			Nflux			2.20	1.62	293.10	36.70	97.15	24.05					Servili 2000 10.17660/ActaHortic.2002.586.116
			enz			1.99	1.43	459.55	40.35	113.95	24.90					Servili 2000 10.17660/ActaHortic.2002.586.116
			Nflux+enz			3.60	2.26	552.60	34.05	177.15	32.60					Servili 2000 10.17660/ActaHortic.2002.586.116
Apulia	2006		O2 0Kpa			6.79	10.00	478.87	144.24	475.59		30.81	8.12			Servili 2008 DOI: 10.1021/jf800694h
Apulia	2006		O2 30Kpa			3.15	5.88	437.70	135.30	361.91		25.83	7.96			Servili 2008 DOI: 10.1021/jf800694h
Apulia	2006		O2 50Kpa			4.40	7.80	343.08	126.16	339.15		29.19	8.64			Servili 2008 DOI: 10.1021/jf800694h
Apulia	2006		O2 100Kpa			1.38	4.35	229.86	125.11	170.61		27.14	7.93			Servili 2008 DOI: 10.1021/jf800694h
Apulia								80.00	111.00	46.00	26.00					Arribas-Lorenzo 2009 DOI 10.1007/s00217-009-1026-z
Apulia			control			1.50	2.90	322.50	107.40	111.60	12.00					Clodoveo 2017 https://doi.org/10.1016/j.ultsonch.2017.01.004
Apulia			control			1.20	3.10	280.00	105.20	131.40	11.80					Clodoveo 2017 https://doi.org/10.1016/j.ultsonch.2017.01.004
Apulia			control			1.80	2.80	306.20	107.20	112.80	13.20					Clodoveo 2017 https://doi.org/10.1016/j.ultsonch.2017.01.004
Apulia			ultrasound			1.80	3.40	387.30	115.50	112.80	12.80					Clodoveo 2017 https://doi.org/10.1016/j.ultsonch.2017.01.004
Apulia			ultrasound			1.90	3.70	384.50	121.80	143.20	13.20					Clodoveo 2017 https://doi.org/10.1016/j.ultsonch.2017.01.004
Apulia			ultrasound			2.00	3.70	401.70	123.60	161.40	15.30					Clodoveo 2017 https://doi.org/10.1016/j.ultsonch.2017.01.004
Apulia			ultrasound			1.80	3.60	389.00	134.00	134.30	13.50					Clodoveo 2017 https://doi.org/10.1016/j.ultsonch.2017.01.004
Apulia			ultrasound			1.60	3.10	366.00	117.70	124.10	13.20					Clodoveo 2017 https://doi.org/10.1016/j.ultsonch.2017.01.004
Apulia Giov			control			2.50	3.70	163.80	76.70	102.30	133.20					Del Coco 2021 https://doi.org/10.1016/j.foodchem.2020.128778
Apulia Giov			ultrasound			0.70	1.20	345.50	90.50	109.30	155.30					Del Coco 2021 https://doi.org/10.1016/j.foodchem.2020.128778
Apulia Sann			control			1.00	1.90	75.70	42.70	66.10	132.90					Del Coco 2021 https://doi.org/10.1016/j.foodchem.2020.128778
Apulia Sann			ultrasound			1.30	1.70	127.00	69.20	91.20	150.90					Del Coco 2021 https://doi.org/10.1016/j.foodchem.2020.128778
Lebanon	2022							109.96	70.26					11.99	3.89	El-Riachy 2023 https://doi.org/10.3390/plants12142681
Balochistan	2016					9.16	14.66	62.35	51.87	1.48	9.16			5.06	2.50	Rizwan 2019 doi : 10.5650/jos.ess18150
Argentina	2014					5.65	6.80	22.94	18.53	31.00	144.40	0.78	0.96	4.44	0.96	Monasterio 2017 DOI: 10.1021/acs.jafc.7b02664
Argentina	2014					29.40	19.62	21.05	9.56	30.40	141.90	0.63	1.04	5.11	1.04	Monasterio 2017 DOI: 10.1021/acs.jafc.7b02664
Uruguay	2014	1m	control			55.80	43.36	37.77	53.85	24.97	9.47					Guerrini 2016 DOI: 10.1111/jfpe.12489
Uruguay	2014	6m	separator			15.24	18.26	22.61	43.98	13.90	9.23					Guerrini 2016 DOI: 10.1111/jfpe.12489
Uruguay	2014	1m	control			57.97	45.33	32.14	55.08	18.83	14.94			1.36	1.07	Guerrini 2016 DOI: 10.1111/jfpe.12489
Uruguay	2014	6m	separator			18.22	20.81	21.34	44.25	17.76	12.08			2.00	1.60	Guerrini 2016 DOI: 10.1111/jfpe.12489
Apulia	2013	1y				17.20	0.50	6.20	10.70	2.30	1.70					Baiano 2014 DOI: 10.17221/79/2013-CJFS
Apulia	2013	2y				16.70	0.50	5.50	10.70	1.40	1.20					Baiano 2014 DOI: 10.17221/79/2013-CJFS
Apulia	2012					0.40	0.50	20.10	16.80	3.50	1.00					Baiano 2013 doi:10.1007/s11746-012-2141-8
Apulia	2006				dec	3.07	2.43	32.21	55.61	10.92						Gambacorta 2010 doi: 10.1111/j.1750-3841.2010.01516.x
Apulia	2006				jan	1.86	1.91	29.72	31.98	6.59						Gambacorta 2010 doi: 10.1111/j.1750-3841.2010.01516.x
Apulia	2006	0m				0.34	0.54	37.11	46.30	6.98						Gambacorta 2010 doi: 10.1111/j.1750-3841.2010.01516.x
Apulia	2006	6m				2.34	2.21	31.38	45.96	10.32						Gambacorta 2010 doi: 10.1111/j.1750-3841.2010.01516.x
Apulia	2006	12m				4.72	3.75	24.40	39.13	8.98						Gambacorta 2010 doi: 10.1111/j.1750-3841.2010.01516.x
Apulia	2006		whole			1.65	1.99	29.19	41.94	7.49						Gambacorta 2010 doi: 10.1111/j.1750-3841.2010.01516.x
Apulia	2006		stoned			3.28	2.34	32.73	45.64	10.02						Gambacorta 2010 doi: 10.1111/j.1750-3841.2010.01516.x
Apulia	2006		45min malax			3.28	2.34	32.73	45.64	10.02						Gambacorta 2010 doi: 10.1111/j.1750-3841.2010.01516.x
Apulia	2006		90min malax			4.98	3.69	29.77	44.78	9.26						Gambacorta 2010 doi: 10.1111/j.1750-3841.2010.01516.x
Apulia		0m	25°C malax			14.90	18.70	39.50	101.00	95.90	62.30			1.40	0.65	Boselli 2009 https://doi.org/10.1016/j.lwt.2008.09.018
Apulia		3m	25°C malax			17.40	7.84	62.00	114.00	48.10	22.10			2.34	0.91	Boselli 2009 https://doi.org/10.1016/j.lwt.2008.09.018
Apulia		6m	25°C malax			28.40	10.40	57.40	86.00	45.60	27.80			1.81	0.75	Boselli 2009 https://doi.org/10.1016/j.lwt.2008.09.018
Apulia		9m	25°C malax			31.80	14.10	19.60	22.80	37.40	8.11			1.43	0.54	Boselli 2009 https://doi.org/10.1016/j.lwt.2008.09.018
Apulia		12m	25°C malax			29.60	11.40	14.90	17.80	35.50	8.11			1.22	0.50	Boselli 2009 https://doi.org/10.1016/j.lwt.2008.09.018
Apulia		0m	35°C malax			17.80	27.10	67.50	132.00	87.30	56.50			1.14	0.53	Boselli 2009 https://doi.org/10.1016/j.lwt.2008.09.018
Apulia		3m	35°C malax			17.10	11.50	89.00	122.00	60.90	26.70			1.66	0.64	Boselli 2009 https://doi.org/10.1016/j.lwt.2008.09.018
Apulia		6m	35°C malax			58.00	35.30	101.00	118.00	58.20	31.50			1.88	0.73	Boselli 2009 https://doi.org/10.1016/j.lwt.2008.09.018
Apulia		9m	35°C malax			52.20	24.10	41.70	39.30	49.90	9.42			1.36	0.52	Boselli 2009 https://doi.org/10.1016/j.lwt.2008.09.018
Apulia		12m	35°C malax			47.50	23.50	22.70	24.00	38.10	7.49			1.17	0.48	Boselli 2009 https://doi.org/10.1016/j.lwt.2008.09.018
Apulia		0m	45°C malax			17.00	17.30	118.00	152.00	62.40	35.50			1.19	0.45	Boselli 2009 https://doi.org/10.1016/j.lwt.2008.09.018
Apulia		3m	45°C malax			9.34	6.88	186.00	176.00	83.50	34.30			1.63	0.57	Boselli 2009 https://doi.org/10.1016/j.lwt.2008.09.018
Apulia		6m	45°C malax			42.20	19.20	113.00	103.00	55.70	26.80			1.16	0.49	Boselli 2009 https://doi.org/10.1016/j.lwt.2008.09.018
Apulia		9m	45°C malax			49.00	21.00	59.70	50.80	53.70	10.40			1.18	0.42	Boselli 2009 https://doi.org/10.1016/j.lwt.2008.09.018
Apulia		12m	45°C malax			48.80	17.80	55.10	46.60	55.00	13.80			1.25	0.46	Boselli 2009 https://doi.org/10.1016/j.lwt.2008.09.018
			Enzyme ABC 10			5.07	5.13	267.50	193.40	366.50						De Faveri 2008 doi:10.1016/j.bej.2008.04.007
			Enzyme AB 10			4.28	5.07	273.10	189.70	339.00						De Faveri 2008 doi:10.1016/j.bej.2008.04.007
			Enzyme AC 10			4.14	6.47	240.60	182.70	322.90						De Faveri 2008 doi:10.1016/j.bej.2008.04.007
			Enzyme BC 10			3.15	4.15	229.00	153.20	309.20						De Faveri 2008 doi:10.1016/j.bej.2008.04.007
			Enzyme A 15			7.20	4.57	272.90	182.40	409.70						De Faveri 2008 doi:10.1016/j.bej.2008.04.007
			Enzyme A 10			4.24	7.84	221.20	153.20	346.20						De Faveri 2008 doi:10.1016/j.bej.2008.04.007
			Enzyme A 5			3.13	8.59	235.20	175.80	352.70						De Faveri 2008 doi:10.1016/j.bej.2008.04.007
			Enzyme B 15			8.18	13.55	244.30	170.80	379.80						De Faveri 2008 doi:10.1016/j.bej.2008.04.007
			Enzyme B 10			6.24	8.63	209.60	150.60	340.50						De Faveri 2008 doi:10.1016/j.bej.2008.04.007
			Enzyme B 5			5.01	5.50	234.10	177.50	295.70						De Faveri 2008 doi:10.1016/j.bej.2008.04.007
			Enzyme C 15			5.40	11.81	221.10	165.70	313.00						De Faveri 2008 doi:10.1016/j.bej.2008.04.007
			Enzyme C 10			5.96	14.66	208.30	171.00	301.00						De Faveri 2008 doi:10.1016/j.bej.2008.04.007
			Enzyme C 5			4.94	7.96	157.00	165.20	275.50						De Faveri 2008 doi:10.1016/j.bej.2008.04.007
			control			2.47	3.94	153.70	148.00	265.40						De Faveri 2008 doi:10.1016/j.bej.2008.04.007
Apulia	2019		control			1.00	2.00	555.30	105.00	126.50	17.40	33.40	15.20			Veneziani 2023 https://doi.org/10.3390/foods12030612
Apulia	2019		O2 0.2L/min			1.30	2.40	527.10	106.30	128.50	21.30	34.20	15.40			Veneziani 2023 https://doi.org/10.3390/foods12030612
Apulia	2019		O2 0.4L/min			1.20	2.50	472.30	105.30	112.70	22.20	33.50	15.00			Veneziani 2023 https://doi.org/10.3390/foods12030612
Apulia	2019		O2 0.8L/min			1.00	2.00	268.10	107.40	146.50	17.60	34.30	13.90			Veneziani 2023 https://doi.org/10.3390/foods12030612
						4.00	5.80	230.20	72.10	221.70		13.70	7.00			Selvaggini 2006 10.1021/jf0527596
						4.30	6.30	216.40	80.00	223.80		22.60	34.10			Selvaggini 2006 10.1021/jf0527596
Apulia	2008					1.97	1.30	66.66	47.84	28.00	7.98	8.80		1.07		Gambacorta 2012 DOI: 10.1002/ejlt.201200043
Apulia	2008					3.96	4.36	22.47	25.76	4.86	2.69	8.37		2.18	1.79	Gambacorta 2012 DOI: 10.1002/ejlt.201200043
Apulia	2008					0.19	0.36	35.57	30.46	5.96	3.87	11.48		1.61		Gambacorta 2012 DOI: 10.1002/ejlt.201200043
						1.61	19.55	57.85	172.47	73.92	40.17					Dinnella 2007 10.1021/jf072244+
							7.13	89.19	187.77	89.00	37.93					Dinnella 2007 10.1021/jf072244+
						16.42	45.59	100.48	253.47	139.16	64.39					Dinnella 2007 10.1021/jf072244+
						4.71	20.89	34.40	137.75	57.96	32.04					Dinnella 2007 10.1021/jf072244+
Marche	2018					10.50	8.20	103.00	64.40	32.40	11.60			5.45	2.05	Di Lecce 2020 https://doi.org/10.3390/foods9070904
Abruzzo	1996					4.54	4.71	787.71	376.49	176.52	55.87					Stefanoudaki 2000 https://doi.org/10.1002/1097-0010(200002)80:3<381::AID-JSFA535>3.0.CO;2-4
Apulia	2018		Control			1.80	4.00	467.90	128.60	128.90	25.70	51.10	29.40			Taticchi 2021 https://doi.org/10.1016/j.foodchem.2020.128369
Apulia	2018		V 20°C			3.90	4.50	692.70	129.40	177.00	26.40	54.30	14.00			Taticchi 2021 https://doi.org/10.1016/j.foodchem.2020.128369
Apulia	2018		V 25°C			2.70	3.80	732.20	131.70	198.50	28.00	58.30	17.00			Taticchi 2021 https://doi.org/10.1016/j.foodchem.2020.128369
Apulia	2018		V 30°C			2.90	4.40	785.60	142.70	205.90	23.10	58.70	20.90			Taticchi 2021 https://doi.org/10.1016/j.foodchem.2020.128369
Apulia	2005					2.26	9.34	69.91	61.86	7.09	4.19					Baiano 2009 doi: 10.1111/j.1750-3841.2009.01072.x
Apulia	2019		Control			2.80	3.10	649.80	149.90	212.10	32.00	39.70	15.10	1.30	1.20	Nucciarelli 2022 https://doi.org/10.1007/s11947-022-02862-9
Apulia	2019		Cold			2.50	3.00	674.30	153.30	202.40	30.80	39.80	15.70	1.10	1.00	Nucciarelli 2022 https://doi.org/10.1007/s11947-022-02862-9
Cordoba	2014									524.00	140.00			0.45	0.18	Miho 2018 https://doi.org/10.1016/j.foodchem.2018.06.002
Cordoba	2015							156.00	202.00	918.00	55.00			1.99	1.66	Miho 2018 https://doi.org/10.1016/j.foodchem.2018.06.002
Apulia						7.20	4.10	70.60	32.20	55.50						Di Giovacchino 2017 DOI: 10.1002/ejlt.201600161
Apulia						4.00	4.40	76.80	35.90	61.20						Di Giovacchino 2017 DOI: 10.1002/ejlt.201600161
Apulia						6.90	4.60	92.90	47.10	69.40						Di Giovacchino 2017 DOI: 10.1002/ejlt.201600161
Apulia						6.10	4.20	73.80	44.30	60.20						Di Giovacchino 2017 DOI: 10.1002/ejlt.201600161
Apulia	2013		control 25°C			4.60	3.60	258.30	35.90	234.10		16.50	9.90			Veneziani 2015 DOI: 10.1021/acs.jafc.5b01666
Apulia	2013		FTC 25°C 15min			3.50	2.80	294.30	37.50	246.20		15.80	9.10			Veneziani 2015 DOI: 10.1021/acs.jafc.5b01666
Apulia	2013		FTC 25°C 20min			2.30	2.80	329.30	40.10	278.20		15.70	9.70			Veneziani 2015 DOI: 10.1021/acs.jafc.5b01666
Apulia	2013		control 30°C			3.50	3.30	305.10	42.50	334.10		14.80	8.90			Veneziani 2015 DOI: 10.1021/acs.jafc.5b01666
Apulia	2013		FTC 30°C 15min			2.90	2.60	336.50	51.00	351.60		15.10	8.00			Veneziani 2015 DOI: 10.1021/acs.jafc.5b01666
Apulia	2013		FTC 30°C 20min			2.80	2.80	370.10	59.30	517.50		16.60	9.10			Veneziani 2015 DOI: 10.1021/acs.jafc.5b01666
			control			3.80	5.70	702.20	134.60	281.20	25.50	47.00	21.40			Leone 2018 DOI: 10.1002/ejlt.201700261
			MW+MS			3.40	4.60	849.80	171.10	359.20	28.30	50.20	27.50			Leone 2018 DOI: 10.1002/ejlt.201700261
			MW (microwave)			2.00	3.90	636.60	180.70	319.80	22.80	47.20	31.90			Leone 2018 DOI: 10.1002/ejlt.201700261
			MS (megasonic)			2.50	4.60	823.50	166.90	356.60	27.80	47.10	24.80			Leone 2018 DOI: 10.1002/ejlt.201700261
Apulia	2022		Control			12.00	10.40	586.60	154.40	165.10	18.50	19.60	12.20			Tamborrino 2023 https://doi.org/10.1016/j.fbio.2023.102706
Apulia	2022		enzymes			10.40	8.70	679.70	175.20	207.30	23.10	21.30	12.30			Tamborrino 2023 https://doi.org/10.1016/j.fbio.2023.102706
Apulia	2022		talc			6.80	5.60	598.10	150.90	182.70	19.30	19.00	11.00			Tamborrino 2023 https://doi.org/10.1016/j.fbio.2023.102706
Apulia	2022		enz+talc			7.80	6.10	670.80	163.90	191.60	21.90	20.50	13.30			Tamborrino 2023 https://doi.org/10.1016/j.fbio.2023.102706
			control			0.30	0.15	68.40	26.70	36.40	2.80	3.50	6.00			Tamborrino 2020 DOI: 10.1002/ejlt.202000129
			destoning			3.00	1.50	140.70	36.90	56.90	3.20	3.90	7.80			Tamborrino 2020 DOI: 10.1002/ejlt.202000129
Apulia	2016				1.07	0.28	0.47	28.26	25.35	4.75	3.54	0.86	10.36	3.65	6.07	Caponio 2018 DOI 10.1002/jsfa.8950
Apulia	2016				1.07	0.28	0.46	28.13	25.70	4.78	3.70	1.02	10.90	3.95	6.41	Caponio 2018 DOI 10.1002/jsfa.8950
Apulia	2016				1.07	0.25	0.47	21.86	23.89	4.43	3.65	1.00	10.05	3.24	6.02	Caponio 2018 DOI 10.1002/jsfa.8950
Apulia	2016				1.39	0.54	0.66	28.47	25.49	4.43	3.30	2.38	12.26	4.14	4.13	Caponio 2018 DOI 10.1002/jsfa.8950
Apulia	2016				1.39	0.58	0.75	24.89	26.51	4.98	3.55	2.61	12.44	4.38	4.36	Caponio 2018 DOI 10.1002/jsfa.8950
Apulia	2016				1.39	0.56	0.76	23.27	26.31	4.60	3.31	2.28	12.09	4.50	4.19	Caponio 2018 DOI 10.1002/jsfa.8950
Apulia	2021		Control			24.10	19.40	257.50	74.30	126.80	17.40	36.10	14.20			Tamborrino 2022 https://doi.org/10.1016/j.ifset.2022.103192
Apulia	2021		PEF 3.2			24.40	23.30	328.30	73.30	140.60	19.40	37.50	13.60			Tamborrino 2022 https://doi.org/10.1016/j.ifset.2022.103192
Apulia	2021		PEF 5.1			27.40	21.00	349.40	79.90	138.60	18.60	35.60	12.80			Tamborrino 2022 https://doi.org/10.1016/j.ifset.2022.103192
			Control			2.60	1.70	420.00	93.50	147.30	12.90	35.80	16.30			Tamborrino 2021 https://doi.org/10.1016/j.lwt.2021.111622
			RSHE			4.20	2.50	636.10	123.20	196.10	14.90	46.20	17.70			Tamborrino 2021 https://doi.org/10.1016/j.lwt.2021.111622
			RSHE-MW			3.10	1.90	551.50	116.70	169.30	14.30	41.40	17.80			Tamborrino 2021 https://doi.org/10.1016/j.lwt.2021.111622
			Control			1.20	2.20	415.40	132.10	141.80	21.20	21.30	14.00			Servili 2019 https://doi.org/10.1016/j.ultsonch.2019.104747
			US 3.5bar			0.90	1.60	467.20	156.80	137.30	24.60	20.10	14.60			Servili 2019 https://doi.org/10.1016/j.ultsonch.2019.104747
Apulia	2018		TR			0.30	0.60	31.90	40.10	9.50	3.60			3.20	1.40	Difonzo 2021 https://doi.org/10.1016/j.lwt.2020.11029
Apulia	2018		MR			0.30	1.00	11.60	30.30	7.10	3.80			2.50	1.50	Difonzo 2021 https://doi.org/10.1016/j.lwt.2020.11029
Apulia	2021		Hammer		1.6	5.20	7.50	527.10	225.80	186.20	43.10	34.50	21.30			Tamborrino 2022 https://doi.org/10.3390/foods11193035
Apulia	2021		Knife		1.6	4.30	7.20	767.20	278.80	246.60	44.30	39.60	23.80			Tamborrino 2022 https://doi.org/10.3390/foods11193035
Apulia	2021		Hammer		2.3	2.00	2.10	484.50	167.60	252.60	30.20	28.40	12.80			Tamborrino 2022 https://doi.org/10.3390/foods11193035
Apulia	2021		Knife		2.3	1.80	1.80	598.20	170.20	289.50	30.30	29.00	12.80			Tamborrino 2022 https://doi.org/10.3390/foods11193035
Cordoba	2019		10 min					545.00	109.00	220.00	84.30			4.70	2.20	Miho 2020 https://doi.org/10.1016/j.foodchem.2020.126183
Cordoba	2019		30 min					1062.00	206.00	167.00	79.50			3.60	1.70	Miho 2020 https://doi.org/10.1016/j.foodchem.2020.126183
Cordoba	2019		60 min					1021.00	236.00	140.00	71.80			2.80	1.40	Miho 2020 https://doi.org/10.1016/j.foodchem.2020.126183
Cordoba	2018							427.00	300.00	512.00	130.00			8.80	6.10	Miho 2021 https://doi.org/10.1016/j.foodchem.2020.128357
Sardinia	2016				1	5.70	11.20	112.00	116.90	268.90	120.80	26.90		1.00	0.60	Deiana 2019b https://doi.org/10.1016/j.foodchem.2019.125243
Sardinia	2016				2	2.70	5.90	65.10	89.40	153.20	68.30	29.30		1.00	0.80	Deiana 2019b https://doi.org/10.1016/j.foodchem.2019.125243
Sardinia	2016				3	2.70	6.60	130.00	153.70	181.00	60.40	32.30		1.60	0.90	Deiana 2019b https://doi.org/10.1016/j.foodchem.2019.125243
Sardinia	2016				4	2.00	5.80	147.70	141.00	94.60	25.40	17.10		2.20	1.20	Deiana 2019b https://doi.org/10.1016/j.foodchem.2019.125243
Sardinia	2015					1.90	10.00	178.70	246.70	62.40	20.50	33.90		2.30	1.20	Deiana 2019a doi:10.1002/ejlt.201800135
Sardinia	2019					9.60	46.00	94.60	156.70	201.30	108.90	37.80	10.00	1.30	1.20	Fancello 2022 https://doi.org/10.1016/j.foodcont.2021.108657

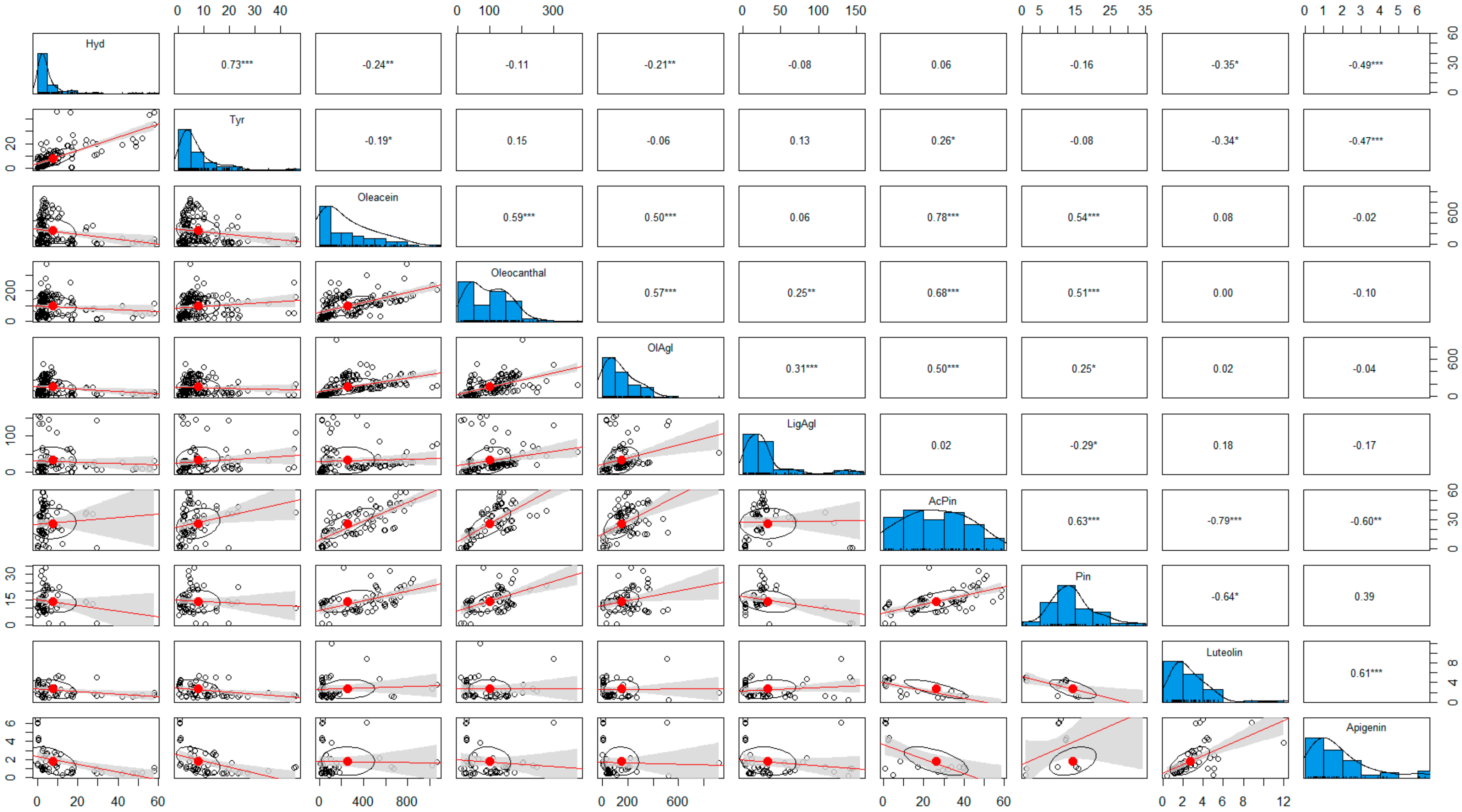

Figure S2. Correlation plot of Coratina phenolic compounds including correlation R values and respective significance, histogram and normal distribution, and data distribution with regression line and confidence intervals.

Table S4. Complete database of Frantoio VOOs phenolic compounds.

Area	Year	Storage	Mill	Irrigation	Canopy	Hyd	Tyr	Oleacein	Oleocanthal	Ol_Agl	LigAgl	Luteolin	Apigenin	AcPin	Pin	Reference
	2005		3ph			1.9	4.6	88.8	43.8	30.3				40.7	4.2	Servili 2007 (10.1021/jf070600)
	2005		stoned			1.8	4.9	112.4	54.8	42.8				42.8	4.5	Servili 2007 (10.1021/jf070600)
Pisa	2008			full		4.0	6.8	180.0	58.0	139.0				28.0	24.0	Caruso 2017 DOI 10.1002/jsfa.8207
Pisa	2008			deficit		3.5	6.8	206.0	76.0	149.0				31.0	25.0	Caruso 2017 DOI 10.1002/jsfa.8207
Pisa	2008			compl		3.2	4.4	223.0	76.0	204.0				22.0	21.0	Caruso 2017 DOI 10.1002/jsfa.8207
Pisa	2008				top	3.7	7.8	252.0	98.0	180.0				27.0	24.0	Caruso 2017 DOI 10.1002/jsfa.8207
Pisa	2008				LS	4.2	5.7	215.0	69.0	170.0				27.0	24.0	Caruso 2017 DOI 10.1002/jsfa.8207
Pisa	2008				LN	2.8	4.5	142.0	44.0	141.0				27.0	22.0	Caruso 2017 DOI 10.1002/jsfa.8207
Pisa	2009			full		3.3	9.2	208.0	72.0	158.0				35.0	28.0	Caruso 2017 DOI 10.1002/jsfa.8207
Pisa	2009			deficit		4.8	8.1	178.0	100.0	192.0				32.0	26.0	Caruso 2017 DOI 10.1002/jsfa.8207
Pisa	2009			compl		1.9	5.7	210.0	130.0	160.0				32.0	26.0	Caruso 2017 DOI 10.1002/jsfa.8207
Pisa	2009				top	4.0	8.1	267.0	126.0	164.0				34.0	30.0	Caruso 2017 DOI 10.1002/jsfa.8207
Pisa	2009				LS	2.8	7.9	217.0	107.0	198.0				31.0	27.0	Caruso 2017 DOI 10.1002/jsfa.8207
Pisa	2009				LN	3.1	7.0	112.0	69.0	149.0				34.0	24.0	Caruso 2017 DOI 10.1002/jsfa.8207
						0.3	10.1	136.5	78.2	98.3				56.3	54.4	Selvaggini 2006 10.1021/jf0527596
						0.4	11.7	158.1	82.8	121.3				58.1	55.1	Selvaggini 2006 10.1021/jf0527596
	2015		3ph			1.2	1.9	146.1	39.0	91.0	4.9			40.6	11.3	Veneziani 2017 https://doi.org/10.1016/j.lwt.2017.09.034
	2015		CT 3ph			0.8	1.6	196.8	49.3	102.2	8.1			40.0	11.2	Veneziani 2017 https://doi.org/10.1016/j.lwt.2017.09.034
Brazil	2012					0.3	2.4					4.2	0.5	31.5	2.5	Ballus 2015 http://dx.doi.org/10.1016/j.foodchem.2014.08.054
Cordoba	2018					2.1		261.0	218.0	317.0	233.0	6.3	2.7			Miho 2021 https://doi.org/10.1016/j.foodchem.2020.128357
Cordoba	2017		10min			1.3		321.0	63.5	479.0	260.0	1.6	0.7			Miho 2020 https://doi.org/10.1016/j.foodchem.2020.126183
Cordoba	2017		30min			1.0		390.0	87.3	417.0	288.0	1.0	0.4			Miho 2020 https://doi.org/10.1016/j.foodchem.2020.126183
Cordoba	2017		60min			0.9		415.0	121.0	415.0	306.0	0.8	0.4			Miho 2020 https://doi.org/10.1016/j.foodchem.2020.126183
Cordoba	2017		Control			0.8		419.0	112.0	422.0	364.0					Miho 2020 https://doi.org/10.1016/j.foodchem.2020.126183
Cordoba	2017		Vacuum			1.0		635.0	106.0	598.0	341.0					Miho 2020 https://doi.org/10.1016/j.foodchem.2020.126183
Argentina	2014					29.7	18.1			11.7	38.3	5.3	1.5	4.0	1.1	Monasterio 2017 DOI: 10.1021/acs.jafc.7b02664
Argentina	2014					6.8	5.8			31.3	123.1	5.8	0.7	2.7	0.6	Monasterio 2017 DOI: 10.1021/acs.jafc.7b02664
Pisa	2012			Full		7.3	5.6	238.0	90.0	166.0				18.0	15.0	Gucci 2019 https://doi.org/10.1016/j.agwat.2018.08.022
Pisa	2012			Deficit 1		8.1	5.5	340.0	89.0	266.0				18.0	14.0	Gucci 2019 https://doi.org/10.1016/j.agwat.2018.08.022
Pisa	2012			Deficit 2		7.8	5.6	274.0	71.0	208.0				18.0	14.0	Gucci 2019 https://doi.org/10.1016/j.agwat.2018.08.022
Pisa	2013			Full		16.8	20.3	495.0	87.0	292.0				16.0	27.0	Gucci 2019 https://doi.org/10.1016/j.agwat.2018.08.022
Pisa	2013			Deficit 1		16.1	18.6	582.0	138.0	505.0				17.0	26.0	Gucci 2019 https://doi.org/10.1016/j.agwat.2018.08.022
Pisa	2013			Deficit 2		11.8	10.8	489.0	109.0	290.0				20.0	25.0	Gucci 2019 https://doi.org/10.1016/j.agwat.2018.08.022
Fiesole	2012					3.2	2.3	116.8	61.7	60.5	15.8	24.4	17.5			Cecchi 2018 https://doi.org/10.1002/jsfa.8841
Fiesole	2012					1.4	2.3	101.2	55.6	57.6	16.9	19.3	6.1			Cecchi 2018 https://doi.org/10.1002/jsfa.8841
Marocco	2012					0.8	4.7	65.7	80.5	30.4	48.3	2.5	0.2	1.9	0.7	Bajoub 2017 https://doi.org/10.3390/ijms18010052
Marocco	2013					0.8	4.1	61.8	103.5	24.5	34.8	3.2	0.4	2.3	0.9	Bajoub 2017 https://doi.org/10.3390/ijms18010052
Umbria	2013					6.8	11.7	229.3	26.5	112.9				28.7	13.9	Famiani 2020 https://doi.org/10.1016/j.scienta.2019.109045
Umbria	2013					5.6	9.9	219.3	22.4	101.5				28.2	12.8	Famiani 2020 https://doi.org/10.1016/j.scienta.2019.109045
Umbria	2013					4.3	6.2	210.1	22.5	102.3				26.5	11.7	Famiani 2020 https://doi.org/10.1016/j.scienta.2019.109045
Umbria	2013					6.3	12.1	204.8	24.0	82.7				27.6	17.1	Famiani 2020 https://doi.org/10.1016/j.scienta.2019.109045
Umbria	2013					6.5	13.3	220.3	23.6	107.0				28.6	13.5	Famiani 2020 https://doi.org/10.1016/j.scienta.2019.109045
Umbria	2013					9.3	11.7	198.3	20.4	92.8				28.6	15.0	Famiani 2020 https://doi.org/10.1016/j.scienta.2019.109045
Umbria	2013					3.0	10.7	207.6	21.0	90.2				26.8	13.2	Famiani 2020 https://doi.org/10.1016/j.scienta.2019.109045
Umbria	2013					7.4	16.4	216.7	19.5	65.7				27.5	16.6	Famiani 2020 https://doi.org/10.1016/j.scienta.2019.109045
Davies	2016					1.7	2.7	21.0	22.0	46.0	39.4	6.5	4.4	7.4	8.1	Olmo-Garcia 2019 https://doi.org/10.1016/j.foodres.2019.108649
Libano	2022							12.5	21.2			4.8	2.2			El-Riachy 2023 https://doi.org/10.3390/plants12142681
Cordoba	2001					4.0	1.9	139.1				3.4	2.0			El-Riachy 2012 https://doi.org/10.5424/sjar/2012102-264-11
Ravenna	2003					15.5	30.0	33.3	20.4	8.2	2.6				10.0	Cerretani 2008 DOI 10.1007/s12078-008-9031-3
Firenze	2020	t1	ctrl			0.9	1.2	24.6	23.1	9.4	5.5	3.7	4.1			Angeloni 2022 https://doi.org/10.1007/s11947-022-02884-3
Firenze	2020	t1	N1			0.9	1.3	47.5	31.8	10.4	7.1	4.2	4.3			Angeloni 2022 https://doi.org/10.1007/s11947-022-02884-3
Firenze	2020	t1	N2			1.3	1.4	66.8	38.9	11.1	4.3	5.6	4.5			Angeloni 2022 https://doi.org/10.1007/s11947-022-02884-3
Firenze	2020	t1	N3			1.1	1.5	87.9	47.7	11.2	4.5	6.7	6.0			Angeloni 2022 https://doi.org/10.1007/s11947-022-02884-3
Firenze	2020	t2	ctrl			1.8	1.6	25.7	26.6	18.1	3.7	8.6	8.4			Angeloni 2022 https://doi.org/10.1007/s11947-022-02884-3
Firenze	2020	t2	N1			2.6	2.0	49.3	35.9	21.9	5.7	11.2	11.2			Angeloni 2022 https://doi.org/10.1007/s11947-022-02884-3
Firenze	2020	t2	N2			2.9	2.1	72.7	44.1	22.2	5.1	11.5	10.3			Angeloni 2022 https://doi.org/10.1007/s11947-022-02884-3
Firenze	2020	t2	N3			2.8	2.2	90.1	54.3	25.9	5.4	12.4	10.8			Angeloni 2022 https://doi.org/10.1007/s11947-022-02884-3
Abruzzo	2020					2.0	6.2	80.8	170.6			3.1	1.1			Flaminii 2023 https://doi.org/10.3390/foods12061292
Abruzzo	2021					5.9	6.3	51.5	76.8			3.3	0.6			Flaminii 2023 https://doi.org/10.3390/foods12061292
Sardinia	2015					0.6	5.2	17.1	70.5	36.9	9.3	3.1	1.8	56.9		Deiana 2019a doi:10.1002/ejlt.201800135
Sardinia	2015					1.2	7.8	11.5	31.8	22.1	5.9	2.8	1.1	23.1		Deiana 2019a doi:10.1002/ejlt.201800135
Oristano	2016					3.6	6.1	73.0	88.5	182.0	113.7	2.3	1.1	37.5		Uniss DiA database
Oristano	2016					3.2	5.7	85.6	100.6	195.8	125.2	2.3	1.1	36.7		Uniss DiA database
Oristano	2016					3.0	6.8	183.7	163.6	121.8	98.4	2.5	1.2	24.2		Uniss DiA database
Oristano	2016					3.1	6.7	179.8	154.4	123.7	82.2	2.5	1.4	21.3		Uniss DiA database
Oristano	2017					4.4	11.0	211.9	163.9	133.9	30.8	3.3	2.0	13.5		Uniss DiA database
Oristano	2017					6.0	28.7	180.6	214.1	204.1	58.3	3.5	2.1	12.7		Uniss DiA database
Oristano	2017					8.6	48.0	94.9	170.6	219.7	79.9	3.4	2.1	5.4		Uniss DiA database
Oristano	2018					3.3	9.6	127.2	117.0	111.7	82.0	1.7	1.1	32.5		Uniss DiA database
Oristano	2018					1.2	5.8	13.5	32.0	35.8	53.4	1.6	1.2	34.8		Uniss DiA database
Balochistan						1.8	3.2	45.8	32.6	1.4	16.3	2.4	1.8			Rizwan 2019 doi : 10.5650/jos.ess18150
Calabria	2015					5.3	5.4	98.4	52.9	27.5	4.4	2.9	1.1			Leporini 2018 doi: 10.9755/ejfa.2018.v30.i7.1743
Calabria	2015					4.6	2.5	50.3	34.9	22.5	3.0	2.2	1.1			Leporini 2018 doi: 10.9755/ejfa.2018.v30.i7.1743
Calabria	2015					1.2	1.1	89.4	37.9	19.0		2.8	1.4			Leporini 2018 doi: 10.9755/ejfa.2018.v30.i7.1743
Calabria	2015					1.7	1.7	56.0	34.6	18.9	1.7	1.9	0.7			Leporini 2018 doi: 10.9755/ejfa.2018.v30.i7.1743
Apulia	2008					1.4	1.1	1.0	30.4	1.4	6.8	1.7	2.0			Gambacorta 2012 DOI: 10.1002/ejlt.201200043
Apulia	2008					0.7	2.2	4.9	9.0	3.6	1.2	1.5	0.5			Gambacorta 2012 DOI: 10.1002/ejlt.201200043
Marche	2018					3.3	4.4	30.9	31.2	23.2	14.7	6.9	1.9			Di Lecce 2020 https://doi.org/10.3390/foods9070904
Apulia	2013					23.0	20.2	12.5	20.1	16.0	5.0					Ragusa 2017 https://doi.org/10.3390/foods6100090
Apulia	2005					0.5	3.9	34.6	24.4	15.0						Baiano 2009 doi: 10.1111/j.1750-3841.2009.01072.x
Cordoba	2015							94.0	256.0	185.0	45.0	2.1	1.3			Miho 2018 https://doi.org/10.1016/j.foodchem.2018.06.002
Cordoba	2023							261.0	218.0	317.0	233.0	6.4	2.7			Tomé-Rodriguéz 2024 https://doi.org/10.1016/j.jfca.2024.106128

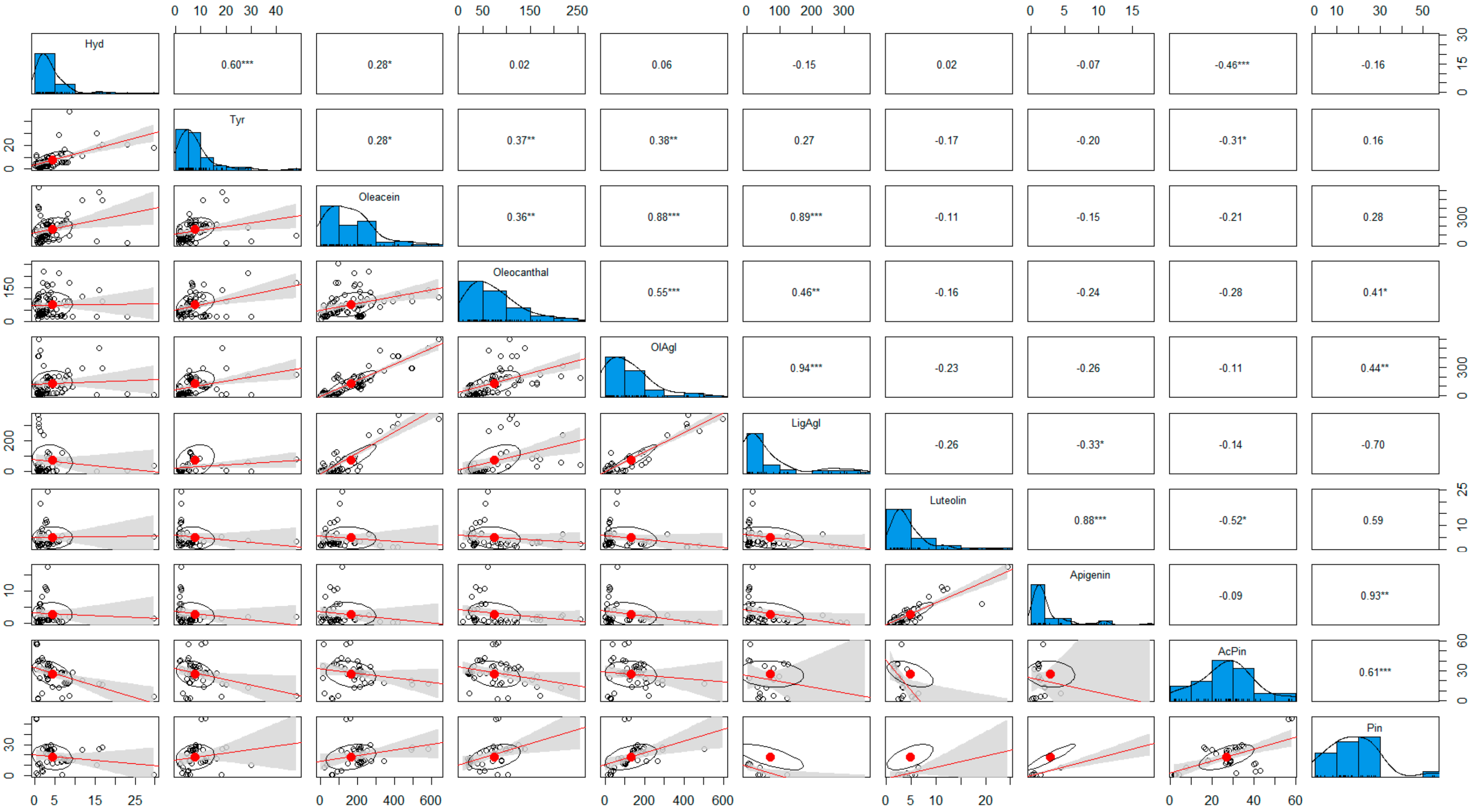

Figure S3. Correlation plot of Frantoio phenolic compounds including correlation R values and respective significance, histogram and normal distribution, and data distribution with regression line and confidence intervals.

Table S5. Results of variance analysis (%) from REML models for three factors of variability (years, growing area, and storage time) affecting VOO phenolic composition.

Variable	*Year*	*Area*	*Storage*	*Residual*
Hydroxytyrosol	30.94	2.02	13.50	**53.54**
Tyrosol	**51.95**	4.13	0.00	43.92
Vanillic acid	20.44	6.13	26.54	**46.89**
p-coumaric acid	**63.55**	17.96	0.00	18.49
Vanillin	**46.90**	13.22	0.00	39.87
Oleacein	0.00	0.00	28.50	**71.50**
Oleocanthal	**46.38**	21.67	7.27	24.67
Acetoxypinoresinol	20.98	0.00	29.53	**49.48**
Oleacein Aglycon	12.06	0.00	2.23	**85.70**
Ligstroside Aglycon	32.04	19.01	6.07	**42.88**
Luteolin	13.08	9.50	**43.32**	34.10
Apigenin	32.22	14.14	20.71	**32.93**
Phenolic alcohols	**50.42**	1.78	0.00	47.80
Phenolic acids	**42.13**	10.98	12.88	34.01
Secoiridoids	31.73	10.65	0.00	**57.61**
Flavonoids	18.81	11.84	**35.60**	33.75
Health Claim	33.20	10.73	0.00	**56.07**
Sum of phenols	33.18	11.31	0.00	**55.51**
Ratio Oleacein-Oleocanthal	21.97	2.95	0.00	**75.08**
Ratio Flavonoids	10.53	12.45	28.04	**48.98**
Ratio Aglycons	17.13	16.34	18.85	**47.68**
Ratio Acids	5.65	14.82	15.62	**63.91**

Table S6. Autofit results for the Orthogonal Projections to Latent Structures Discriminant Analysis (OPLS-DA) models.

Model name	***Type***	***R^2X***	***R^2Y***	***Q^2Y***	***n. Components***	***cv-ANOVA p-value***
Year	OPLS-DA	0.824	0.645	0.537	3+2+0	0.000
Area	OPLS-DA	0.625	0.410	0.276	3+0+0	0.034
Storage	OPLS-DA	0.653	0.750	0.685	1+2+0	0.000
